# Eukaryotic initiation factor 4 A-3 promotes glioblastoma growth and invasion through the Notch1-dependent pathway

**DOI:** 10.1186/s12885-023-10946-8

**Published:** 2023-06-15

**Authors:** Lei Wei, Mika Pan, Qiulan Jiang, Beiquan Hu, Jianyi Zhao, Chun Zou, Liechun Chen, Chunhai Tang, Donghua Zou

**Affiliations:** 1 Department of Neurology, The Fifth Affiliated Hospital of Guangxi Medical University, Nanning, 530022 Guangxi China; 2 Department of Neurology, The Second Affiliated Hospital of Guangxi Medical University, Nanning, 530007 Guangxi China; 3 Department of Radiation Oncology, The Affiliated Hospital of Youjiang Medical University for Nationalities, Baise, 533000 Guangxi People’s Republic of China; 4 Department of Neurosurgery, The Fifth Affiliated Hospital of Guangxi Medical University, Nanning, 530022 Guangxi China; 5 Department of Neurosurgery, RenJi Hospital, Shanghai Jiao Tong University School of Medicine, Shanghai, 200127 China; 6 Department of Neurosurgery, The Second Affiliated Hospital of Guangxi Medical University, Nanning, 530007 Guangxi China; 7 The Second Affiliated Hospital of Guangxi Medical University, No. 166 Daxue Dong Road, Nanning, 530007 Guangxi China

**Keywords:** EIF4A3, Glioblastoma, Invasion, Migration, Notch, Prognosis, Proliferation

## Abstract

**Background:**

As an adult tumor with the most invasion and the highest mortality rate, the inherent heterogeneity of glioblastoma (GBM) is the main factor that causes treatment failure. Therefore, it is important to have a deeper understanding of the pathology of GBM. Some studies have shown that Eukaryotic Initiation Factor 4A-3 (EIF4A3) can promote the growth of many people’s tumors, and the role of specific molecules in GBM remains unclear.

**Methods:**

The correlation between the expression of EIF4A3 gene and its prognosis was studied in 94 GBM patients using survival analysis. Further in vitro and in vivo experiments, the effect of EIF4A3 on GBM cells proliferation, migration, and the mechanism of EIF4A3 on GBM was explored. In addition, combined with bioinformatics analysis, we further confirmed that EIF4A3 contributes to the progress of GBM.

**Results:**

The expression of EIF4A3 was upregulated in GBM tissues, and high expression of EIF4A3 is associated with poor prognosis in GBM. In vitro, knockdown of EIF4A3 significantly reduced the proliferation, migration, and invasion abilities of GBM cells, whereas overexpression of EIF4A3 led to the opposite effect. The analysis of differentially expressed genes related to EIF4A3 indicates that it is involved in many cancer-related pathways, such as Notch and JAK-STAT3 signal pathway. In Besides, we demonstrated the interaction between EIF4A3 and Notch1 by RNA immunoprecipitation. Finally, the biological function of EIF4A3-promoted GBM was confirmed in living organisms.

**Conclusion:**

The results of this study suggest that EIF4A3 may be a potential prognostic factor, and Notch1 participates in the proliferation and metastasis of GBM cells mediated by EIF4A3.

**Supplementary Information:**

The online version contains supplementary material available at 10.1186/s12885-023-10946-8.

## Introduction

Glioblastoma (GBM) is dismal tumor with survival rarely exceeding 12 months after diagnosis despite of multimodal therapies [1, 2]. Due to the aggressive and invasive nature of GBMs, they account for only 1.4% of cancers but have a mortality rate of 2.9% [3]. Current treatment for GBM includes surgical resection followed by concurrent radiation therapy and chemotherapy. Unfortunately, molecular heterogeneity between and within tumors [4–6] has led to poor results in many clinical trials [7], with almost all patients with GBM experiencing recurrence months after surgical resection [8, 9]. This underscores the need to identify the biological mechanisms involved in GBM cell proliferation or invasion, which translates into a significant improvement in patient prognosis. In recent years, several biomarkers have been shown to be associated with the prognosis of GBM [10–12]. In addition, some artificial intelligence approaches have shown superiority in promoting targeted clinical applications in GBM [13], however, more molecular evidence is needed to reveal the pathological pattern of GBM.

Eukaryotic initiation factor 4 A-3 (EIF4A3) is a member of the DEAD-box RNA decapacitating enzyme family, which is found mainly in the nucleus and is a core component of the exon junction complex (EJC) [14]. EIF4A3 plays an important role in RNA metabolism, including the roles of nonsense-mediated RNA decay (NMD) and RNA splicing [15, 16]. During EJC splicing, the spliced mRNAs shows higher stability and translation [17, 18]. EIF43A plays an important role in various malignancies, including colorectal, gastric, hepatocellular, and breast cancers [19–22]. In addition, EIF4A3 has been found to promotes malignant biological processes in GBM cells [23], which lays the foundation for an in-depth molecular and mechanistic study of EIF4A3 in GBM.

Notch signaling pathways are evolutionarily conserved pathways involved in cell proliferation, death, activation of differentiation processes, and acquisition of specific cell fates [24, 25]. Aberrant regulation of Notch signaling plays a crucial role in the development of disease. In particular, Notch1 has been shown to be oncogenic in the development of GBM [4, 26]. However, to date, the relevance of EIF4A3 and Notch1 to signal transducer and activator of transcription 3 (STAT3) pathways has not been clearly elucidated in GBM.

Accordingly, we designed a series of in vivo and in vitro functional and molecular assays to explore how EIF4A3 overexpression mediates GBM development and concluded that EIF4A3 is a potential prognostic factor.

## Results

### High expression of EIF4A3 mediates poor prognosis in GBM

**Fig. 1:**
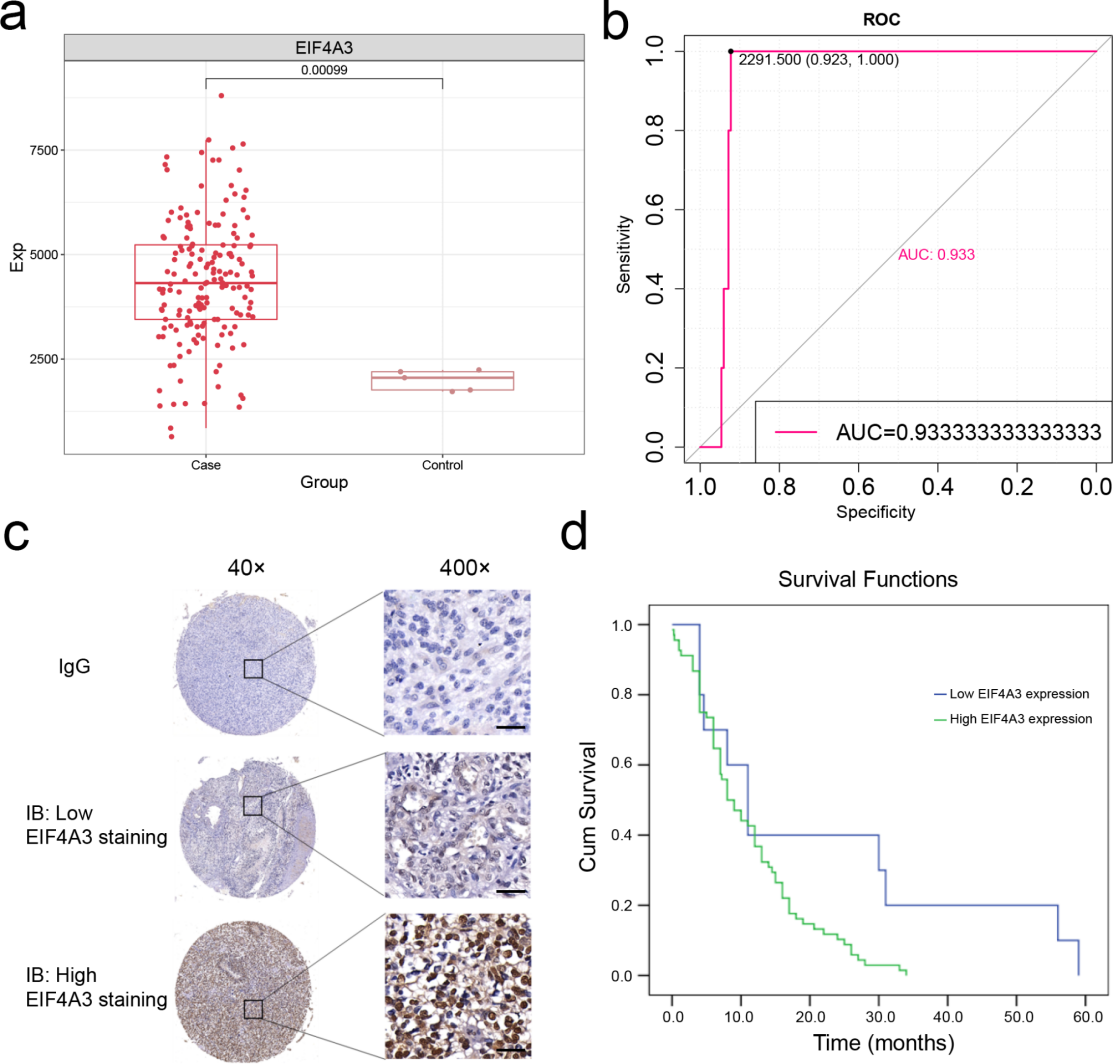
Upregulation of EIF4A3 expression levels suggests a poor prognosis in GBM. (**a**) EIF4A3 was up-regulated in GBM based on The Cancer Genome Atlas. (**b**) The receiver operating characteristic (ROC) curve of EIF4A3 as a biomarker of GBM in The Cancer Genome Atlas. (**c**) EIF4A3 expression in tumor sections of different GBM patients with immunoglobulin as control and magnification. (**d**) High expression of EIF4A3 mediates poor prognosis in GBM patients

First of all, in The Cancer Genome Atlas (TCGA)-GBM data, EIF4A3 is significantly highly expressed in GBM (Fig. 1a). In addition, the receiver operating characteristic (ROC) curve shows that EIF4A3 has excellent diagnostic performance, area under the curve (AUC) = 0.933 (Fig. 1b). Next, using immunostaining to examine the expression of EIF4A3 in GBM specimens from 94 patients (Fig. 1c), we found low and high EIF4A3 expressions in patients with low- and high-grade malignancies, respectively. In addition, we obtained follow-up data from patients and included these patients in the survival analysis. Survival analysis revealed a worse prognosis for GBM patients with high EIF4A3 expression (P < 0.05) (Fig. 1d).

### EIF4A3 regulates cell proliferation in GBM

**Fig. 2:**
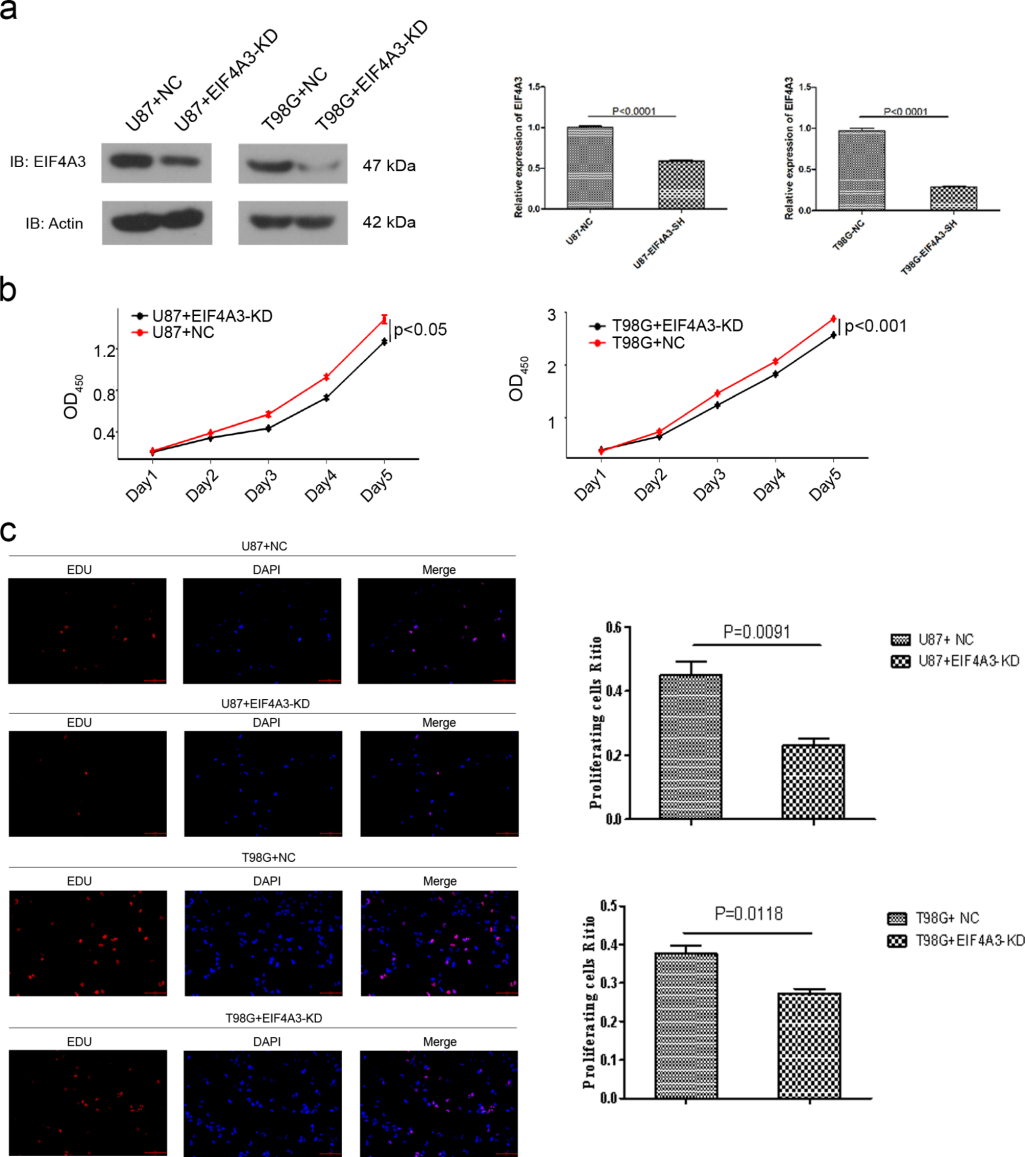
Effect of EIF4A3 knockdown on the proliferation of T98G and U87-MG cells. (**a**) The expression of EIF4A3 in U87-MG and T98G cells after EIF4A3 knockdown (EIF4A3-KD) was determined by Western blotting. β-actin was used as an experimental control. (**b**) The proliferation of EIF4A3-KD and NC-treated U87-MG and T98G cells. (**c**) Proliferation of T98G and U87-MG cell lines in EIF4A3-KD and NC as determined by 5-ethynyl-2′-deoxyuridine (EdU). Scale bar: 20 μm

To elucidate the effects of EIF4A3 in GBM, western blotting method for the determination of EIF4A3 protein levels after knockdown of EIF4A3 in U87-MG and T98G cell lines (Fig. 2a) and overexpression of EIF4A3 in A172 and U251-MG cells with low EIF4A3 expression (Figure S1a), obtaining stable EIF4A3 knockout (EIF4A3-KD) and EIF4A3 overexpressing (EIF4A3-OE) cell lines. Cell Counting Kit-8 (CCK-8) analysis showed that EIF4A3-KD inhibited the proliferative capacity of GBM cells compared to the negative control (NC) (Fig. 2b), whereas EIF4A3-OE enhanced the proliferation of GBM cells (Figure S1b). In addition, the same results were found in the 5-ethynyl-2′-deoxyuridine (EdU) proliferation assay (Fig. 2c, Figure S1c). These findings above confirm that high expression of EIF4A3 promotes GBM proliferation.

### EIF4A3 regulates the migration and invasion of GBM cells

**Fig. 3:**
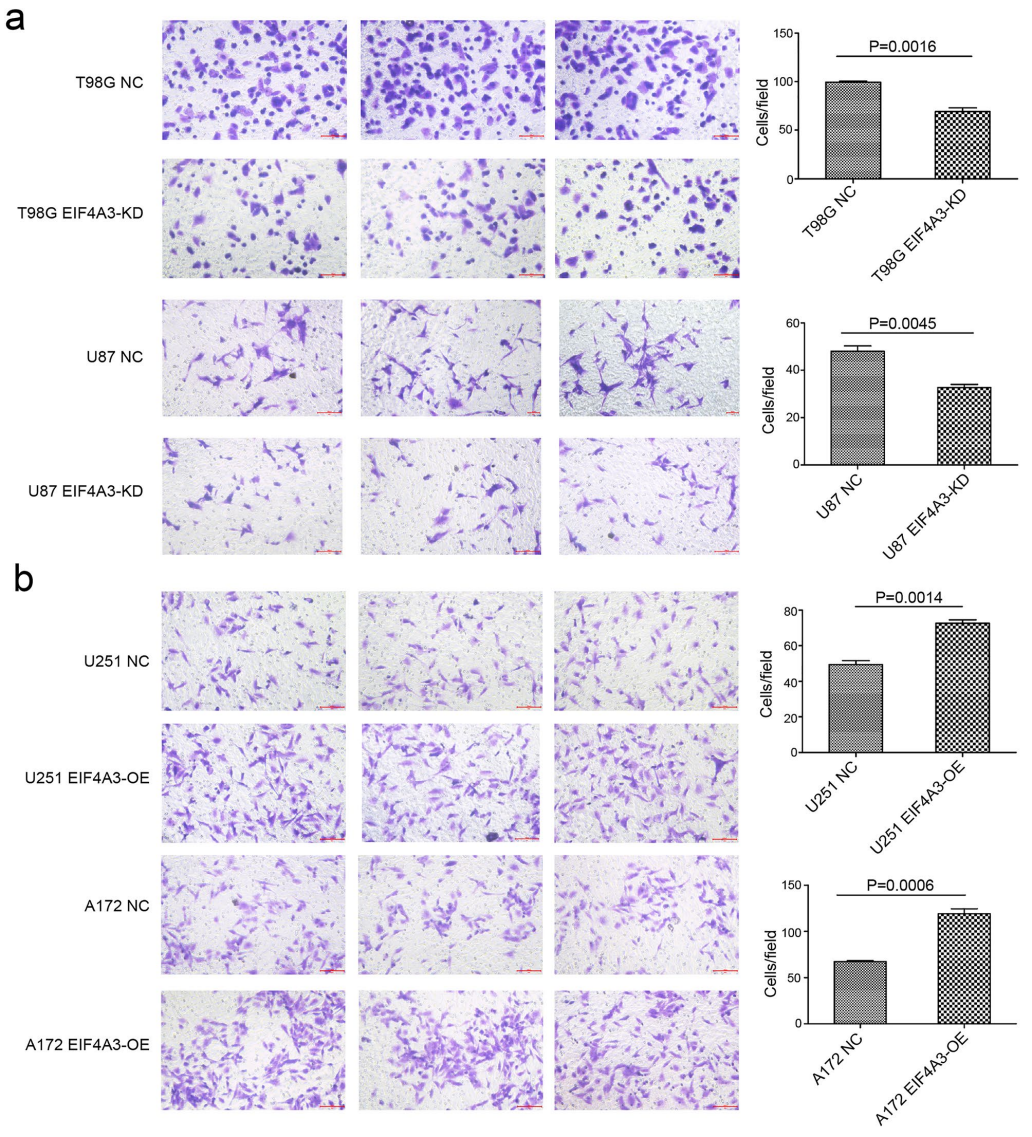
Effect of EIF4A3 knockdown or overexpression on the migratory capacity of GBM cells. (**a**) Migration ability of U87-MG and T98G cells treated with EIF4A3 knockdown (EIF4A3-KD) and negative control (NC). Scale bar: 20 μm. (**b**) EIF4A3 overexpression (EIF4A3-OE) and migration ability of NC-treated U251-MG and A172 cells. Scale bar: 20 μm

We then investigated the migratory and infiltrative effects of EIF4A3 on GBM cells in vitro. It was found by transwell experiments that the migration of cells was significantly and markedly reduced after knockdown of EIF4A3 in T98G and U87-MG cell lines (Fig. 3a). The opposite result was observed after overexpression of EIF4A3 in U251-MG and A172 cells (Fig. 3b). Further, Matrigel-transwell invasion assay showed that the invasive ability of cells was significantly reduced after knockdown of EIF4A3 in T98G and U87-MG cell lines (Figure S2a), while the invasive ability of cells was significantly increased after overexpression of EIF4A3 in A172 and U251-MG cells (Figure S2b).

### EIF4A3 may regulate Notch1 expression through the STAT3-related pathway

**Fig. 4:**
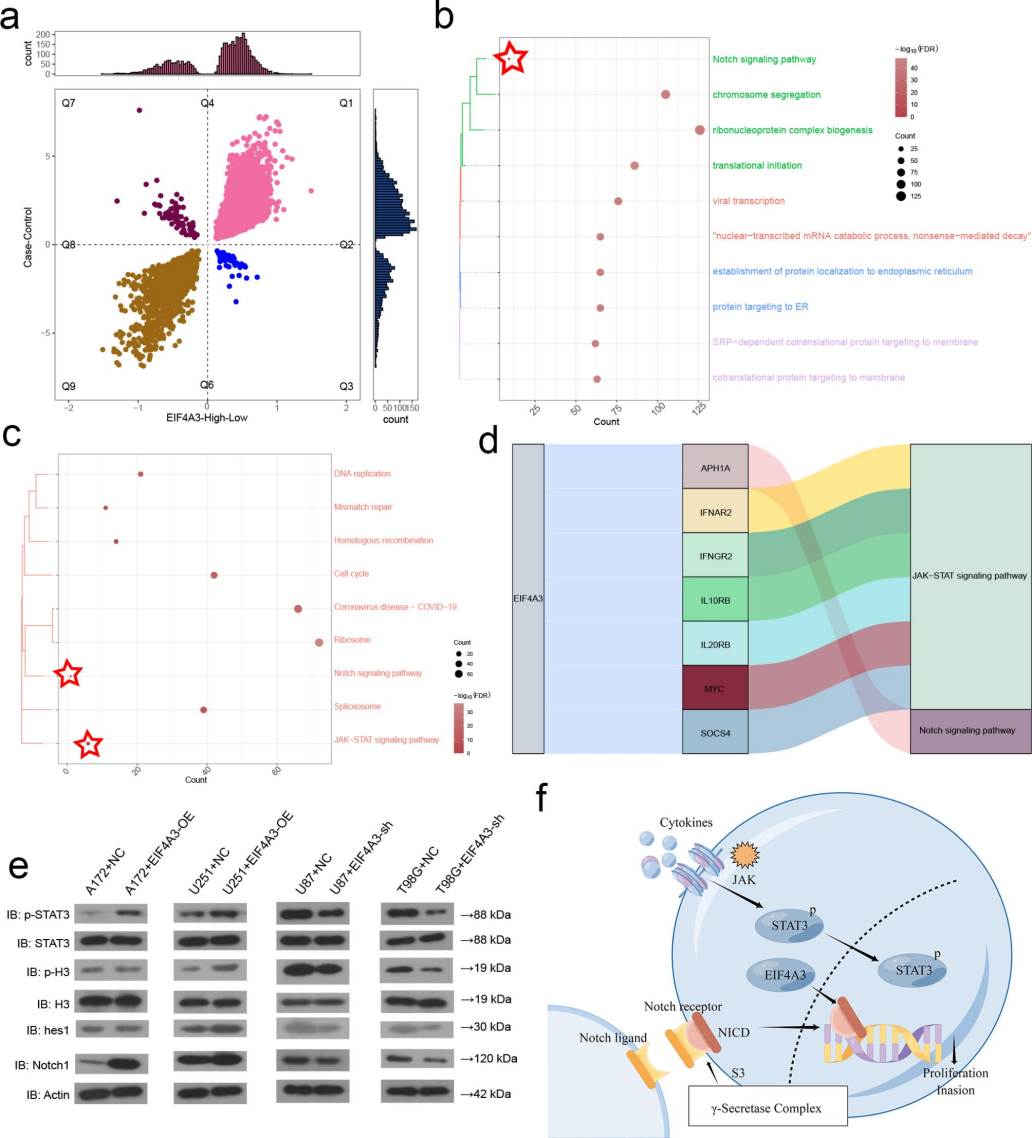
Effect of high EIF4A3 expression on signal transducer and activator of STAT3-related pathways in GBM cells. (**a**) Quadrant plot. The genes in quadrant 1 were upregulated genes consistent with EIF4A3 expression. (**b**) Biological process (BP) enrichment analysis was carried out for the continuously up-regulated genes. (**c**) Analysis of Kyoto Encyclopedia of Genes and Genomes (KEGG) pathway enrichment was performed on the consistently up-regulated genes. (**d**) EIF4A3 regulates Notch1 and STAT3 biological pathways. (**e**) Protein expression levels of STAT3-related signaling pathway genes in A172 and U251-MG cells with EIF4A3 overexpression (EIF4A3-OE) and in U87-MG and T98G cells with EIF4A3 knockdown (EIF4A3-KD). (**f**) EIF4A3 may regulate Notch signaling pathway and JAK − STAT signaling pathway to promote the migration and invasion of GBM cells

To further explore how high EIF4A3 expression regulates the development of GBM, we analyzed the differential gene expression between GBM and control samples, as well as the high or low expression level of EIF4A3. The gene expression consistent with high EIF4A3 was identified as differentially expressed genes (DEGs) (Fig. 4a). These DEGs are mainly involved in Notch signal pathway and JAK-STAT3 signal pathway (Fig. 4b-c). Based on STRING database, we identified the genes that interact with Notch1 and STAT3 (Fig. 4d). We verified the levels of phosphorylated STAT3 (p-STAT3), phosphorylated H3 (p-H3), hes1, and Notch1 in cells overexpressing or knocking down EIF4A3 by western blotting (Fig. 4e), and the levels of these four proteins in EIF4A3 overexpressing A172 and U251-MG cell lines significantly increased, but significantly decreased in the EIF4A3 knockdown T98G and U87-MG cell lines. In addition, the presence of EIF4A3 binding to Notch1 in T98G and U87-MG cell lines was confirmed by the RNA immunoprecipitation (RIP) assay (Figure S3). These results suggest that the up-regulation of EIF4A3 may promote the migration and invasion of GBM cells through Notch signaling pathway (Fig. 4f), the specific mechanism is unclear and needs further research.

### EIF4A3 regulates cell proliferation, migration, and invasion through a Notch1-dependent pathway

**Fig. 5:**
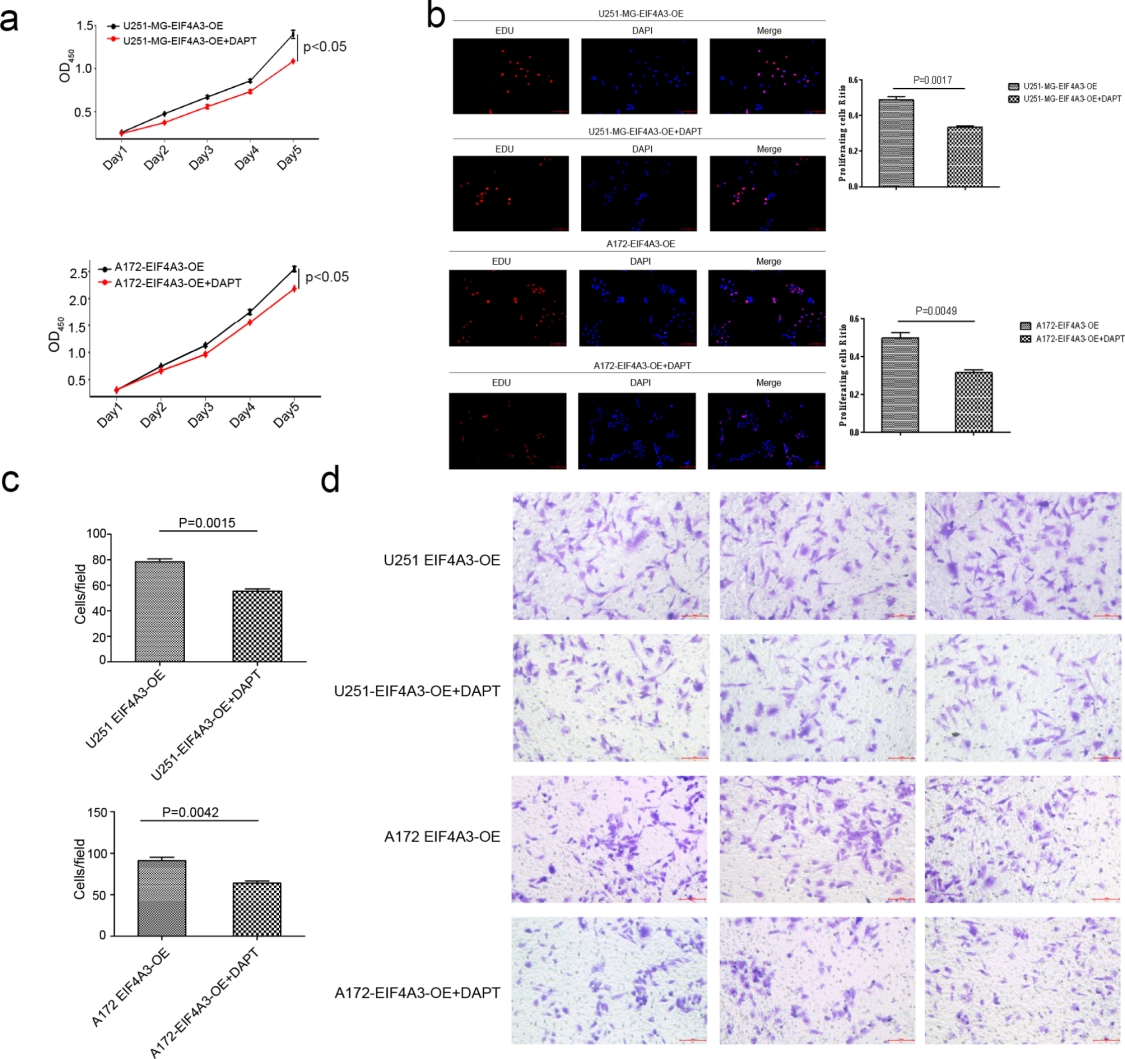
Notch1 is involved in EIF4A3-mediated GBM cell proliferation and migration. (**a**) The proliferation of U251-MG and A172 cells overexpressing EIF4A3 was analyzed in the presence of a NC and DAPT. (**b**) Similarly, the proliferation of the cells was verified, showing representative fluorescence micrographs. Scale bar: 20 μm. (**c-d**) Transwell analysis of blank control and DAPT. Scale bar: 20 μm

The γ-secretase inhibitor DAPT was used to inhibit the Notch pathway in EIF4A3 overexpressing cell lines U251-MG and A172 to explore the association between EIF4A3 and Notch1. The results showed that the proliferation ability of EIF4A3 overexpressing U251-MG and A172 cells treated with DAPT was significantly decreased (Fig. 5a and b). In addition, the migration ability of EIF4A3 overexpressing U251-MG and A172 cells treated with DAPT was significantly reduced (Fig. 5c and d). Simultaneously, we also found that the invasive ability of DAPT-treated EIF4A3 overexpressing U251-MG and A172 cells significantly decreased (Figure S4). These results indicate that DAPT can reverse the proliferation, migration and invasion of EIF4A3 overexpressing cell lines by inhibiting the Notch pathway.

### EIF4A3 regulates GBM-xenograft growth through a Notch1-dependent pathway

**Fig. 6:**
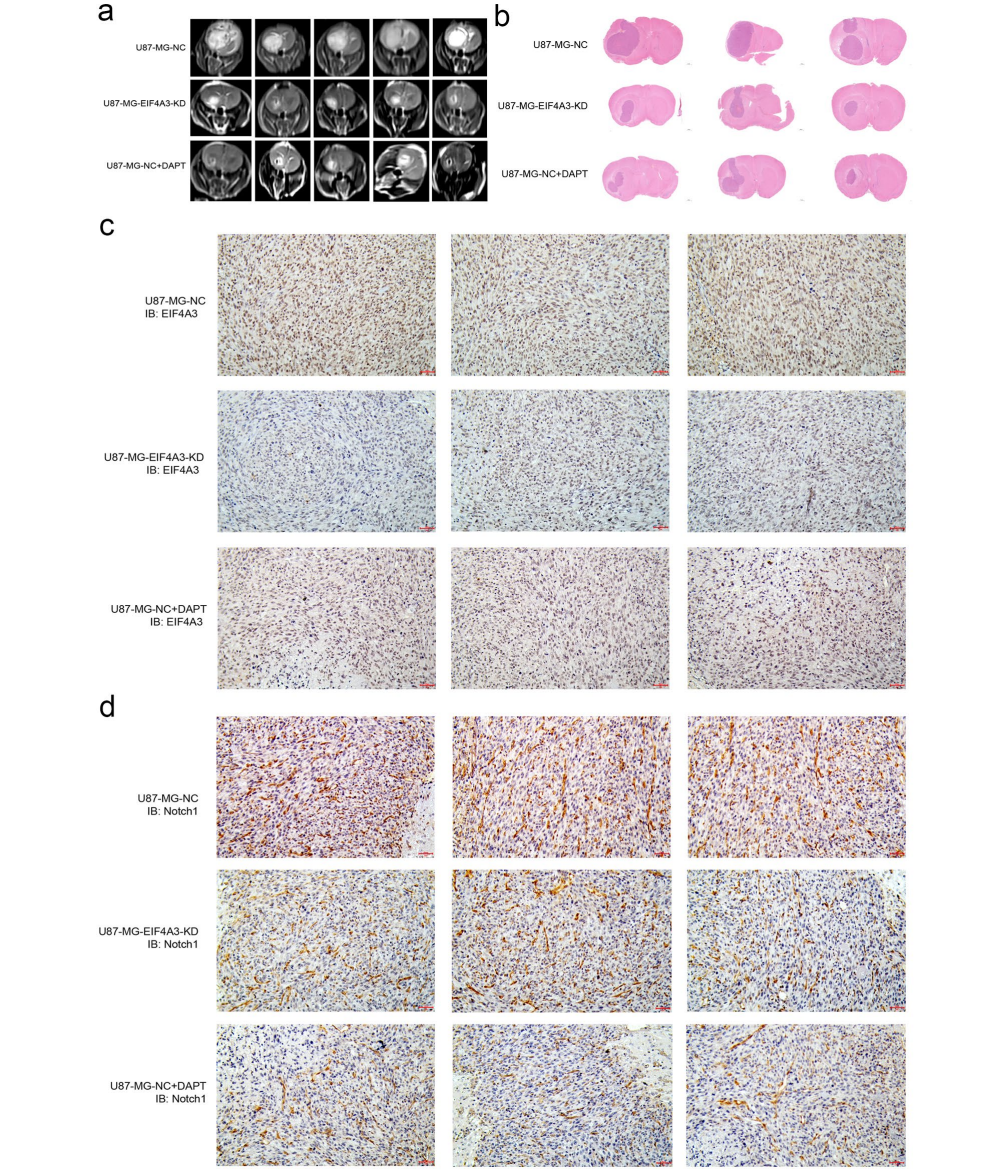
Effect of reduced EIF4A3 on GBM progression in GBM xenografts. (**a**) Intracranial injection of lentivirus-transformed U87-MG cells with NC or EIF4A3-KD into nude mice and U87-MG-NC cells with DAPT added. Animals were examined by magnetic resonance imaging. (**b**) Representative micrographs of hematoxylin-eosin-stained tumor sections. (**c-d**) Immunohistochemistry of tumor tissues stained with anti-EIF4A3 and anti-Notch1 antibody. Scale bar: 20 μm

The brain was inoculated with U87-NC and U87-EIF4A3-KD tumor cells in situ to establish a glioma cell transplantation model in situ, whereas DAPT was co-incubated with U87-NC for 48 h. The DAPT-treated cells were injected into nude mice, and the size of intracranial tumors in nude mice was observed by magnetic resonance imaging (MRI). Nude mice had smaller tumors (Fig. 6a and b). In addition, EIF4A3-KD or the addition of DAPT significantly reduced the levels of EIF4A3 and Notch1 proteins in the tissues (Fig. 6c and d). DAPT treated U87-NC cells, and western blotting was used to detect the expression level of EIF4A3 in U87-NC cells before and after treatment. The results showed that there was no significant change in the expression of EIF4A3 after adding DAPT (Figure S5).

## Discussion

In this study, we confirmed that EIF4A3 could promote the proliferation and invasion of GBM cells by regulating Notch1 through in vivo and in vitro experiments.

In the present study, through in vivo and in vitro experiments, we confirm that EIF4A3 induces proliferation, migration, and invasion of GBM. Although EIF4A3 deficiency has been demonstrated to impede cell migration and impair cell viability [27, 28], the exact mechanism that allows excessive cell death following reduced EIF4A3 expression in GBM is elusive and is an interesting question addressed in our current study.

Some studies have confirmed that EIF4A3 can enhance the proliferation and metastasis of gastric cancer [20]. In triple-negative breast cancer, E2F1 and EIF4A3 promoted high expression of circRNA circSEPT9, which in turn promoted cell proliferation, invasion, tumorigenesis, and metastasis in vivo [22]. Moreover, EIF4A3-induced circ_0084615 promoted colorectal cancer proliferation, migration, and invasion via the miR-599/ONECUT2 axis [29]. Consistent with the present study, our study confirmed that high expression of EIF4A3 promotes proliferation, migration and invasion of GBM.

RIP analysis also demonstrated that EIF4A3 binds to Notch1. The EIF4A3 inhibitor DAPT significantly inhibited the proliferation and invasion of GBM cells, and this inversion was caused by the downregulation of Notch1 gene expression. Notch1 gene expression is closely associated with the development of GBM, and high levels of Notch1 protein expression mediate the poor prognosis of GBM patients [30–32]. Experiments by Li et al. confirmed that Notch1 knockout U251-MG cells xenografted into mice cranially died later relative to the control group [33]. Conversely, over-activated Notch1 may promote differentiation and vascularization of glioma cells, which in turn accelerates their invasion and metastasis [34]. In the Notch signaling pathway, the most commonly targeted genes are the HES family of transcriptional repressors [35]. The hairy enhancer of cleavage 1 (Hes1) is an important target gene downstream of the Notch1 signaling pathway, which is involved in the proliferation of GBM [31, 36, 37]. This experiment was similarly confirmed in the present study. In contrast, the progression of GBM by linking EIF4A3 to Notch1 has not yet been explored. Although EIF4A3 has been confirmed to bind to Notch1 by RIP analysis in our experiments, the upstream regulation and downstream targets of the Notch signaling pathway in GBM should be further investigated in the future to clarify the pathogenesis of GBM and to develop more optimal therapeutic strategies.

## Materials and methods

### Patients

Specimens used in this study were obtained from 94 patients with GBM who underwent surgical resection at RenJi Hospital, Shanghai Jiao Tong University School of Medicine between January 2005 and December 2013. All included patients were pathologically confirmed as GBM, independently reviewed by two pathologists, and classified according to the World Health Organization (WHO) tumor grade criteria. All procedures involving human-derived samples in this study were approved by the Ethics Committee of RenJi Hospital, Shanghai Jiao Tong University School of Medicine, and samples were obtained from all patients after they or their legal guardians signed the appropriate written consent forms.

### Data processing

TCGA (https://www.cancer.gov/) were downloaded from the UCSC Xena browser (http://xena.ucsc.edu/public), including gene expression profiles based on Affymetrix Human Genome U133a array platform (Affymetrix; Thermo Fisher Scientific, Waltham, MA, USA) and clinical information. We used the limma package in R to identify the DEGs between the GBM sample and the healthy brain tissue in TCGA-GBM [38] as well as the EIF4A3 high-low expression level (the critical value is the median of EIF4A3 expression) between the GBM samples. The difference related to P < 0.05 (adjusted by error detection rate) is considered significant. The gene consistent with the high expression of EIF4A3 is considered to be the GBM specific gene related to EIF4A3. Finally, use pROC package to analyze ROC curve to determine whether EIF4A3 may become a diagnostic marker of GBM [39].

### Immunohistochemistry

Sections of tumor and normal tissue samples from GBM patients were subjected to immunohistochemistry, and sections were independently assessed for EIF4A3 expression in the sections by two pathologists with no knowledge of the patients’ clinicopathological information.

### Lentiviral vector-mediated knockdown of EIF4A3

Human-derived GBM cell lines U87-MG, U251-MG, A172, and T98G purchased from the American Typical Culture Collection (ATCC). To select stable EIF4A3-KD cell lines and NC cell lines, small hairpin RNA (shRNA) encoding targeting human EIF4A3 was constructed by Hanyin (Shanghai, China), and in addition lentiviral expression plasmids encoding green fluorescent protein (GFP) were used as negative controls for infection of U87-MG and T98G cells. The knockdown effect was examined using real-time quantitative polymerase chain reaction (RT-qPCR) and western blotting techniques.

### Lentivirus-Mediated overexpression of EIF4A3

An expression plasmid encoding FLAG-tagged EIF4A3 (Hanin) was constructed and the corresponding empty vector was transfected into 293T cells as NC. U251-MG and A172 cells were infected with these recombinant retroviruses with an infection plural of 1 to obtain EIF4A3-OE cells or NC cells.

### Functional enrichment analysis

To explore the biological processes (BPs) and pathways in which the marker for each cell cluster is involved, the R package clusterProfiler was used to perform Gene Ontology (GO) and Kyoto Encyclopedia of Genes and Genomes (KEGG) enrichment analyses [40, 41], with P < 0.05 being considered to indicate significance.

### Western blotting analysis

We detected the levels of p-STAT3, STAT3, p-H3, H3, hes1, Notch1, and β-actin in GBM cell lines by western blotting [31, 42–44].

### Cell proliferation analysis

Cell proliferation was detected using the CCK-8 (Beyotime, Shanghai, China). In addition, proliferation assays based on EdU were performed according to the manufacturer’s instructions (Invitrogen, Carlsbad, California, USA).

### Transwell analysis

Cells were inoculated into the upper chamber of a 24-well Transwell plate (Millipore, Billerica, MA, USA) with or without a Matrigel-coated membrane. The insert was placed in the bottom chamber of a 24-well plate containing dulbecco’s modified Eagle’s medium (DMEM) and 10% fetal bovine serum (FBS) as a chemoattractant. 24 h later, the number of cells in five random areas of each chamber was counted and averaged by staining the bottom layer of invading cells with 1% crystal violet and imaged with a digital microscope.

### RNA immunoprecipitation

T98G and U87-MG cells were collected and lysed in RIP lysis buffer, immunoprecipitated with antibodies to protein A/G magnetic beads and measured by RT-qPCR.

### Xenograft animal models

Thymus-free nude mice (n = 6) were cultured until 6 weeks of age, and nude mice were anesthetized and implanted intracranially into the striatum with NC, EIF4A3-KD, or U87-MG cells combined with DAPT using a small animal stereotactic rack (David Kopf Instruments, Tujunga, California, USA), and tumor size was monitored by MRI.

Tumors were collected after execution of nude mice, paraffin embedded, paraffin sections were made, and the effect of EIF4A3 on tumor histomorphology was observed by hematoxylin-eosin (HE) staining, and the expression levels of EIF4A3 and Notch1 were detected by immunohistochemistry.

### Statistical analyses

Survival analysis of patients with high and low expression of EIF4A3 was performed using the Kaplan-Meier method [45]. In addition, for the bioinformatics analysis of TCGA data, we use the Bioinforcloud platform (http://www.bioinforcloud.org.cn). All statistical analyses were performed using the R package [46] and Statistical Package for the Social Sciences (SPSS) for Windows version 17.0 (SPSS, Chicago, Illinois, USA). A two-tailed P value was considered significant when it was less than 0.05.

## Conclusions

Our study provides rich and compelling evidence that Notch1 is involved in EIF4A3-mediated GBM cell proliferation, migration, and invasion. Finally, we confirmed the involvement of EIF4A3 and Notch1 in tumorigenesis in living organisms. Thus, our study may reveal that EIF4A3 can serve as a promising prognostic biomarker or therapeutic target in GBM treatment. However, there are inherent limitations to the experimental model in our study. It is only a preliminary exploration of the role of EIF4A3 and Notch1 in GBM. Future studies should include the establishment of knockout mouse models to validate the role of EIF4A3 and Notch1 in GBM development in animal experiments.

## Electronic supplementary material

Below is the link to the electronic supplementary material.

Supplementary Material 1

Supplementary Material 2

## Data Availability

All data that supports the findings of this study are available in this published article and the supplementary information files.
