# Supplementary material for: Eukaryotic initiation factor 4 A-3 promotes glioblastoma growth and invasion through the Notch1-dependent pathway
Source: BMC Cancer. 2023 Jun 15;23:550. doi: 10.1186/s12885-023-10946-8 (PMC10273507; doi:10.1186/s12885-023-10946-8)
Supplement: Supplementary file 1 — Supplementary Material 1 [file 12885_2023_10946_MOESM1_ESM.pdf]

Supplementary Materials:

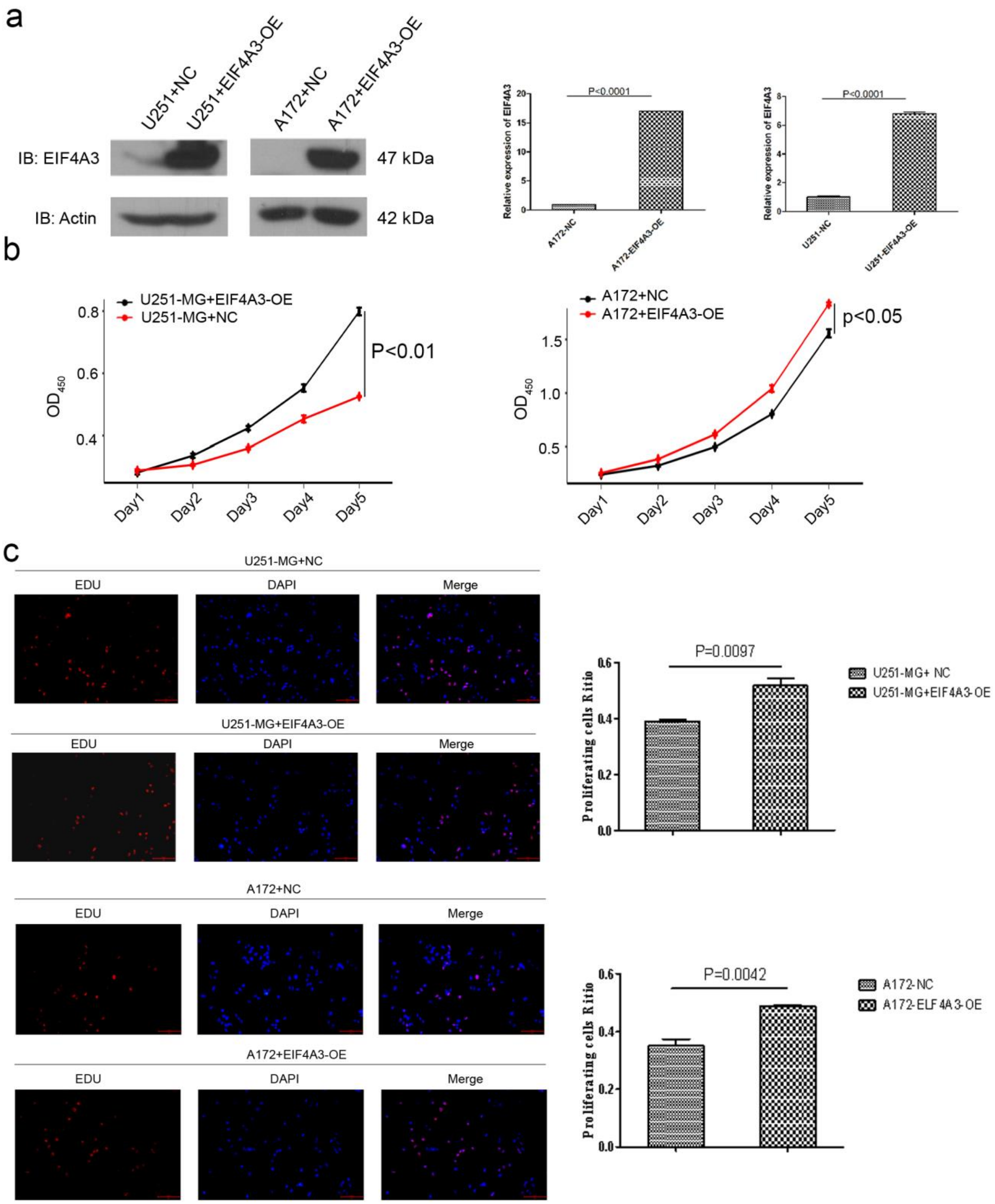

Figure S1. Effect of EIF4A3 overexpression on the proliferation of U251-MG and A172 cells.

(**a**) Expression of EIF4A3 in U251-MG and A172 cells transfected with control lentivirus (NC) or lentivirus overexpressing EIF4A3 (EIF4A3-OE) was assessed by protein blotting. (**b**) Proliferation of GBM cell lines U251-MG and A172 with EIF4A3-OE and NC was analyzed using CCK8 assay. (**c**) Representative fluorescence micrographs are shown in the GBM cell lines U251-MG and A172 of EIF4A3-OE and NC, where cell proliferation was determined using the EdU assay. Scale bar: 20μm.

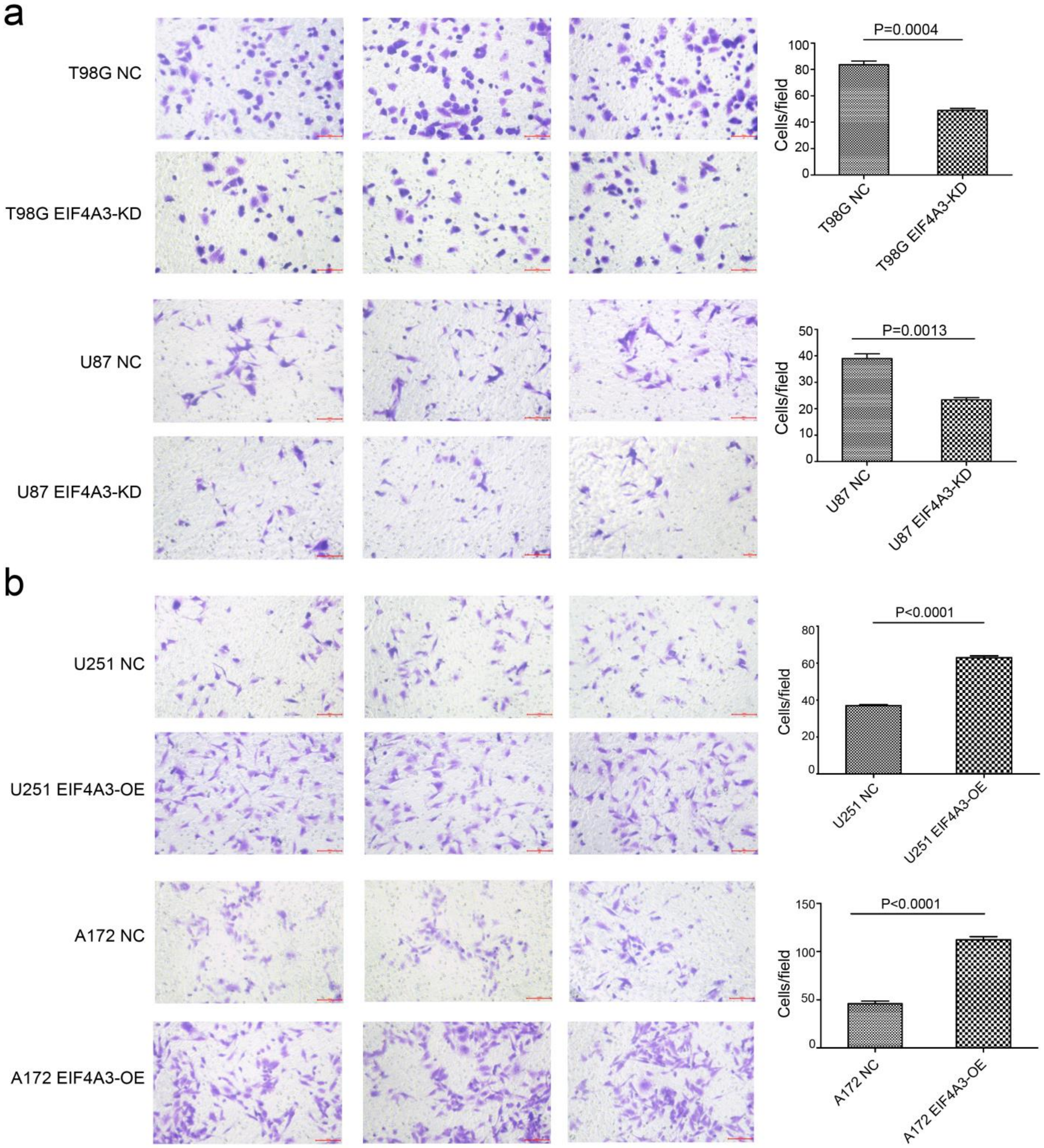

Figure S2. Effect of EIF4A3 knockdown or overexpression on the invasive capacity of GBM cells.

(**a**) T98G and U87-MG cells were treated with EIF4A3 knockdown (EIF4A3-KD) and negative control (NC), and the invasive ability of the cells was assayed using Transwell assay with stromal gel. Scale bar: 20μm. (**b**) U251-MG and A172 cells were treated with EIF4A3 overexpression (EIF4A3-OE) and negative control (NC), and the invasive ability of the cells was detected using Transwell assay with stromal gel. Scale bar: 20μm.

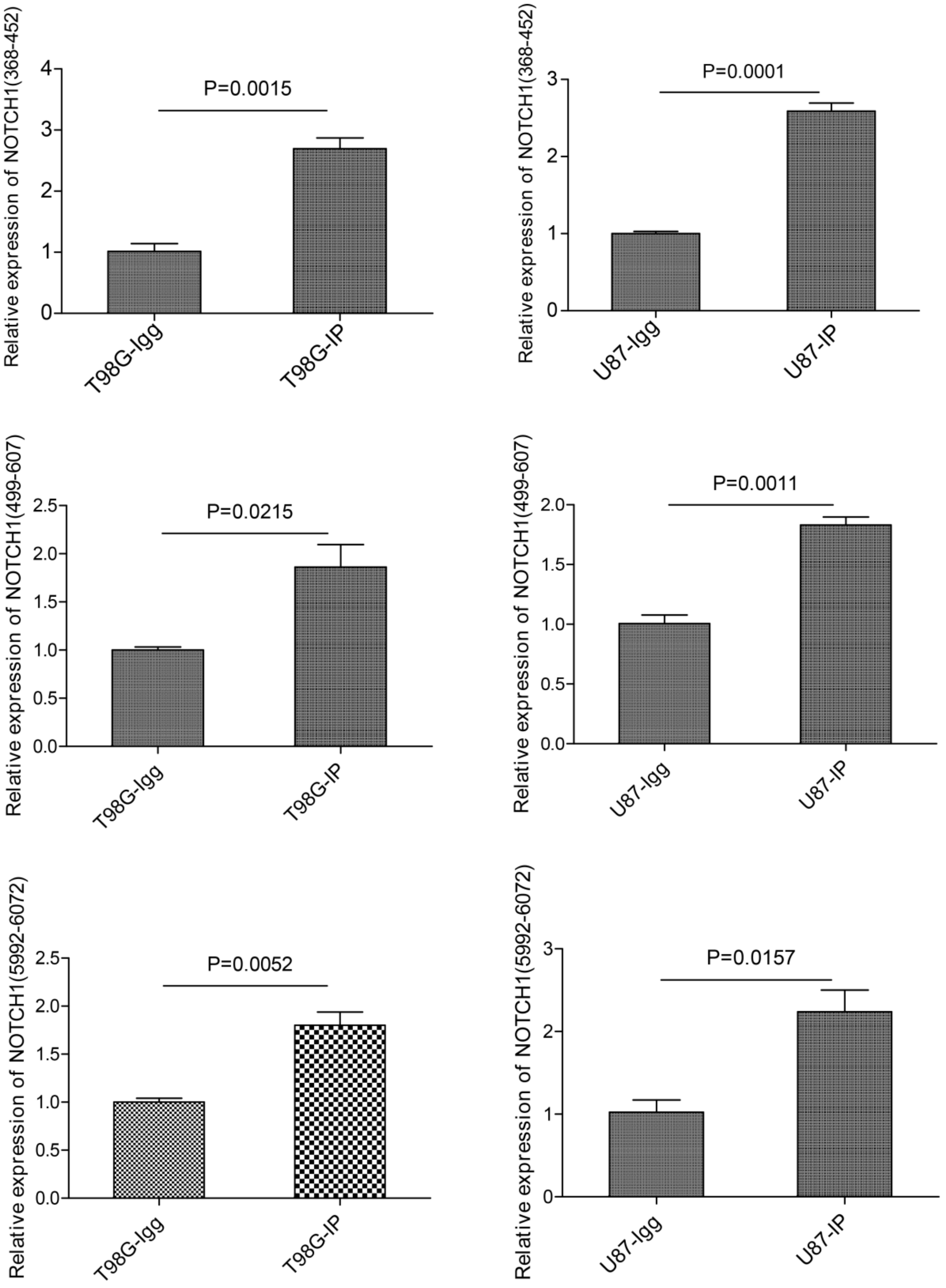

Figure S3. Reciprocal relationship between EIF4A3 and Notch1.

After RIP of EIF4A3 antibody against T98G and U87-MG cells, the possibility of binding between EIF4A3 and different Notch1 fragments was detected.

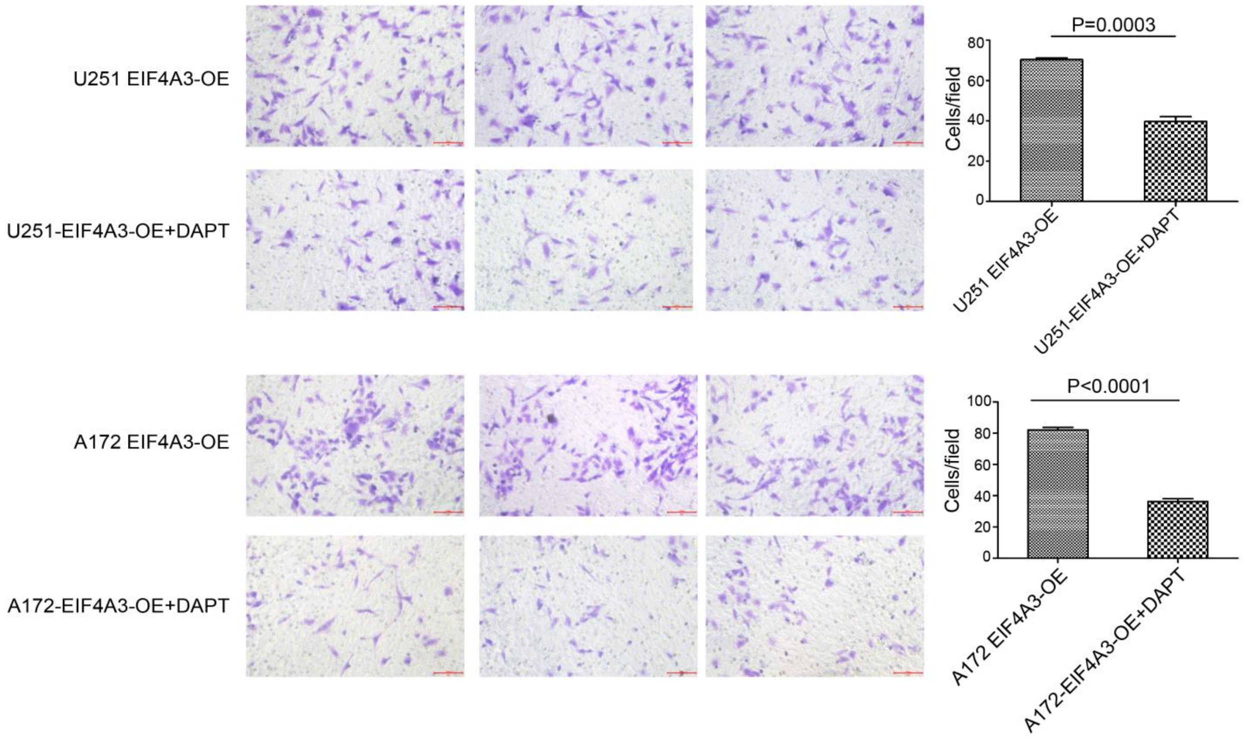

Figure S4. Notch1 is involved in EIF4A3-mediated GBM cell invasion.

Invasion ability of U251-MG and A172 cells overexpressing EIF4A3 was analyzed using Matrigel-Transwell in the presence of blank control and γ-secretase inhibitor DAPT. Scale bar: 20μm.

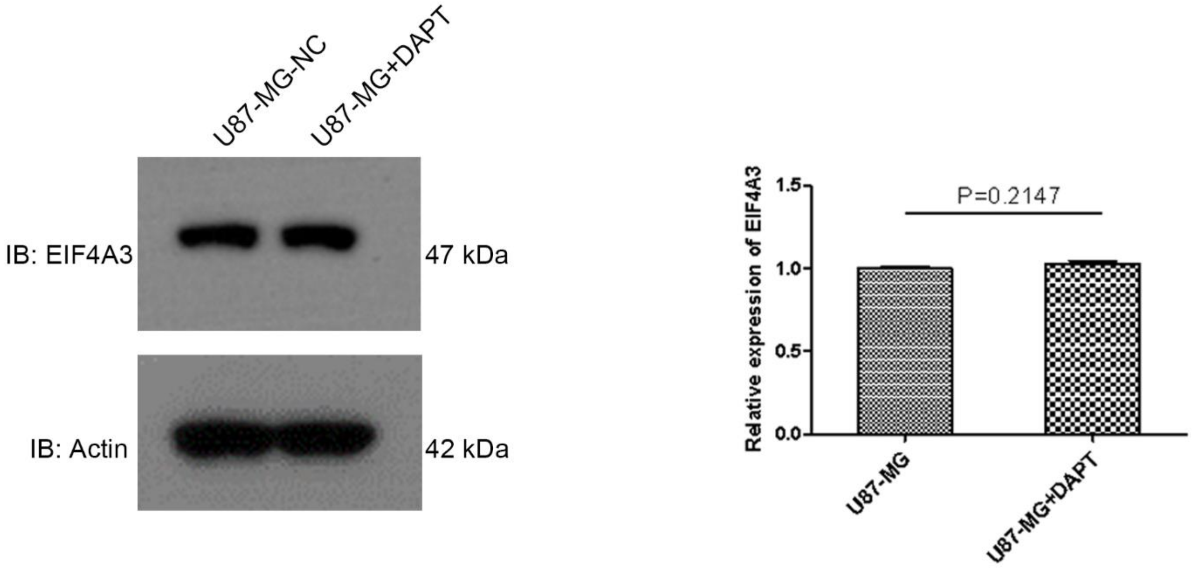

Figure S5. DAPT treated U87-NC cells, and WB was used to detect the expression level of EIF4A3 in U87-NC cells before and after treatment.

The results showed that there was no significant change in the expression of EIF4A3 after adding DAPT.
