# Supplementary material for: Eukaryotic initiation factor 4 A-3 promotes glioblastoma growth and invasion through the Notch1-dependent pathway
Source: BMC Cancer. 2023 Jun 15;23:550. doi: 10.1186/s12885-023-10946-8 (PMC10273507; doi:10.1186/s12885-023-10946-8)

Original data of Figure 2b:

	U87+NC			U87+EIF4A3-KD		
**Day1**	0.212	0.222	0.215	0.205	0.209	0.207
**Day2**	0.384	0.387	0.398	0.347	0.344	0.339
**Day3**	0.523	0.594	0.592	0.456	0.428	0.421
**Day4**	0.910	0.915	0.959	0.723	0.706	0.759
**Day5**	1.543	1.499	1.404	1.270	1.246	1.286

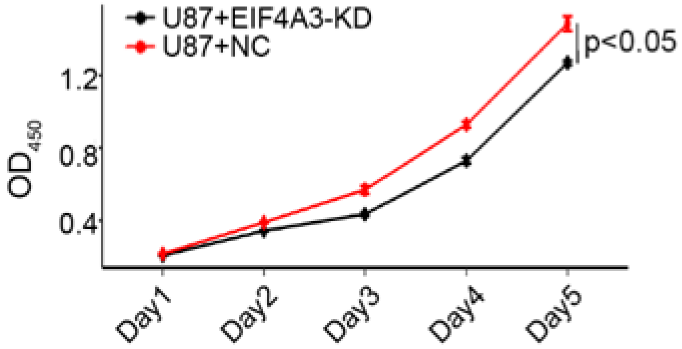

	T98G+NC			T98G+EIF4A3-KD		
**Day1**	0.359	0.373	0.368	0.379	0.398	0.387
**Day2**	0.719	0.716	0.772	0.671	0.612	0.652
**Day3**	1.461	1.447	1.480	1.213	1.243	1.257
**Day4**	2.089	2.019	2.095	1.824	1.860	1.790
**Day5**	2.875	2.880	2.875	2.572	2.555	2.580

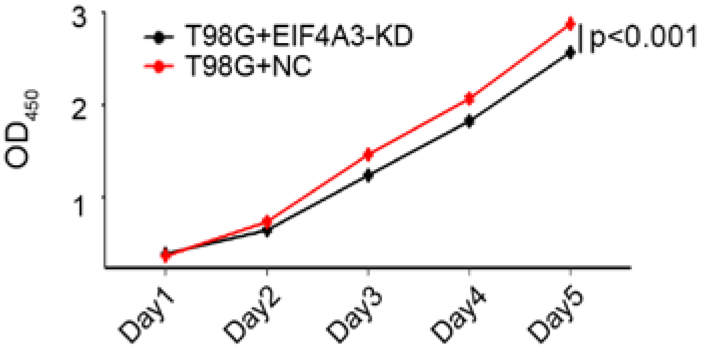

Original data of Figure 5a:

	U251-MG-EIF4A3-OE			U251-MG-EIF4A3-OE+DAPT		
Day1	0.263	0.259	0.254	0.245	0.244	0.250
Day2	0.473	0.478	0.476	0.366	0.365	0.389
Day3	0.646	0.666	0.698	0.598	0.563	0.513
Day4	0.839	0.846	0.889	0.700	0.743	0.757
Day5	1.489	1.333	1.370	1.095	1.083	1.078

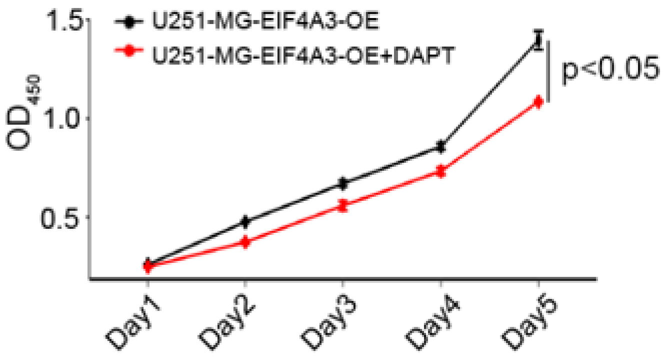

	A172-EIF4A3-OE			A172-EIF4A3-OE+DAPT		
Day1	0.297	0.310	0.310	0.314	0.315	0.312
Day2	0.738	0.739	0.760	0.665	0.687	0.636
Day3	1.169	1.102	1.123	0.983	0.924	0.992
Day4	1.738	1.834	1.684	1.554	1.539	1.581
Day5	2.513	2.485	2.638	2.180	2.190	2.185

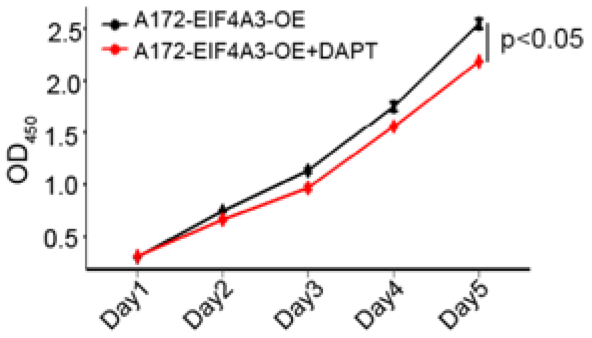

Original data of Figure S1b:

	U251-MG+NC			U251-MG+EIF4A3-OE		
Day1	0.290	0.282	0.295	0.294	0.273	0.281
Day2	0.307	0.302	0.312	0.325	0.349	0.336
Day3	0.353	0.364	0.363	0.414	0.437	0.421
Day4	0.473	0.453	0.434	0.575	0.548	0.535
Day5	0.522	0.527	0.527	0.811	0.811	0.774

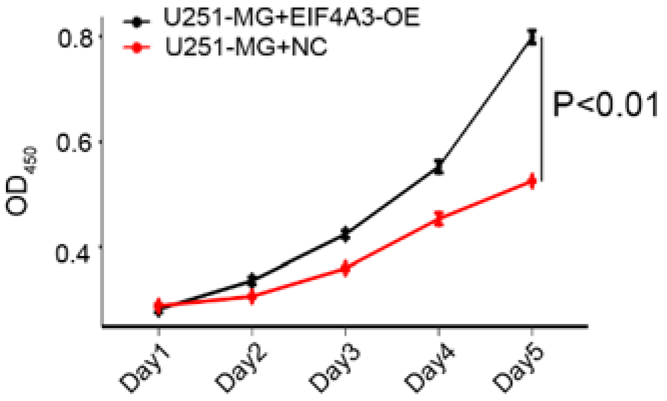

	A172+ NC			A172+EIF4A3-OE		
Day1	0.237	0.238	0.243	0.257	0.250	0.254
Day2	0.333	0.305	0.329	0.383	0.383	0.384
Day3	0.489	0.498	0.504	0.596	0.627	0.622
Day4	0.797	0.803	0.814	1.019	1.102	1.004
Day5	1.531	1.508	1.632	1.810	1.858	1.825

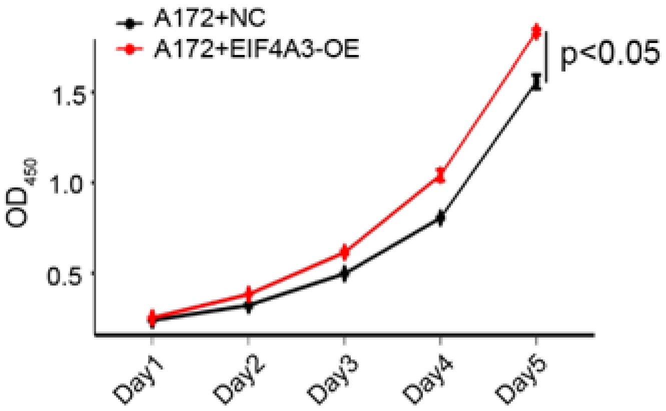

Figure 2a-U87, IB: EIF4A3:

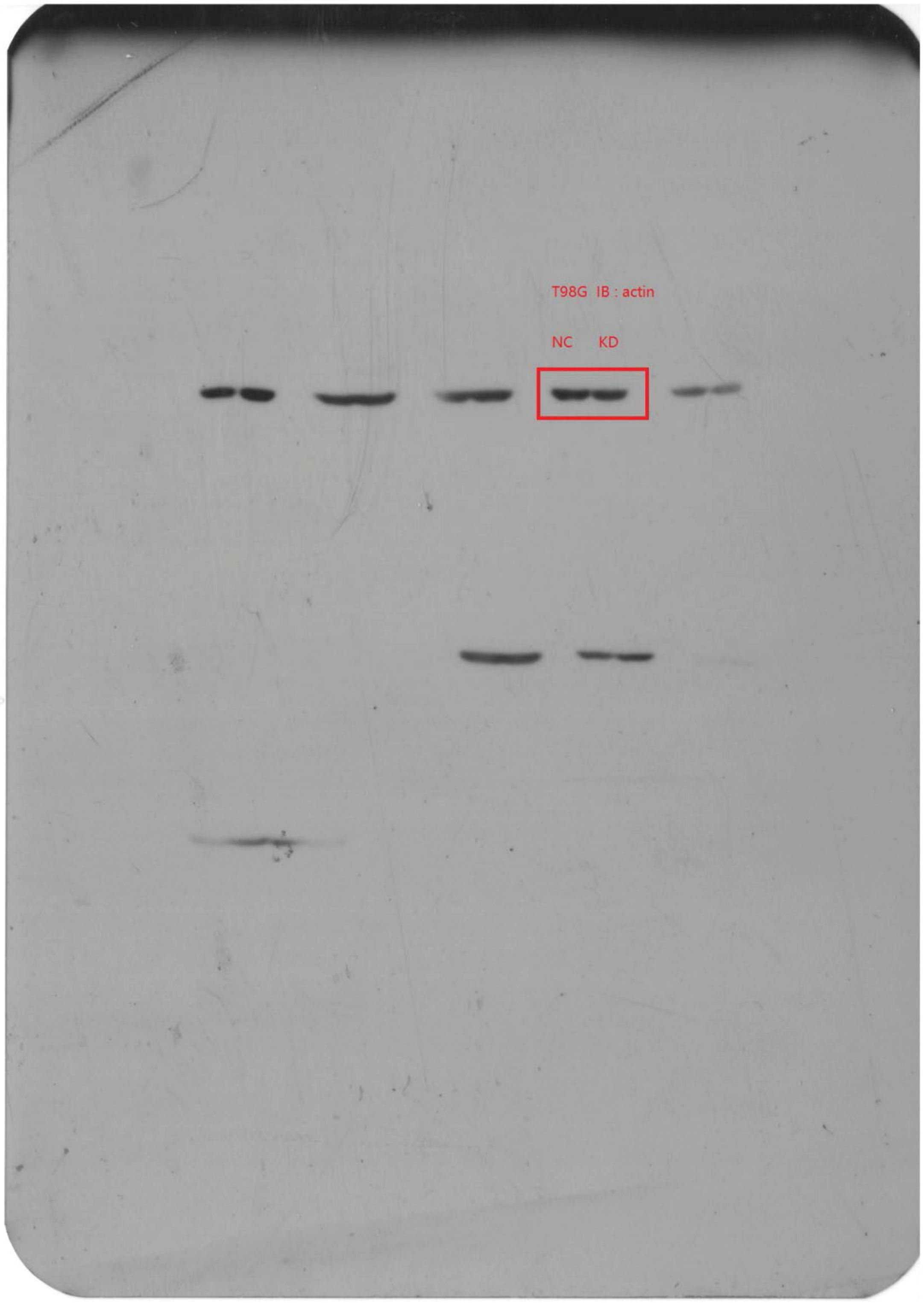

Figure 2a-T98g, IB: EIF4A3:

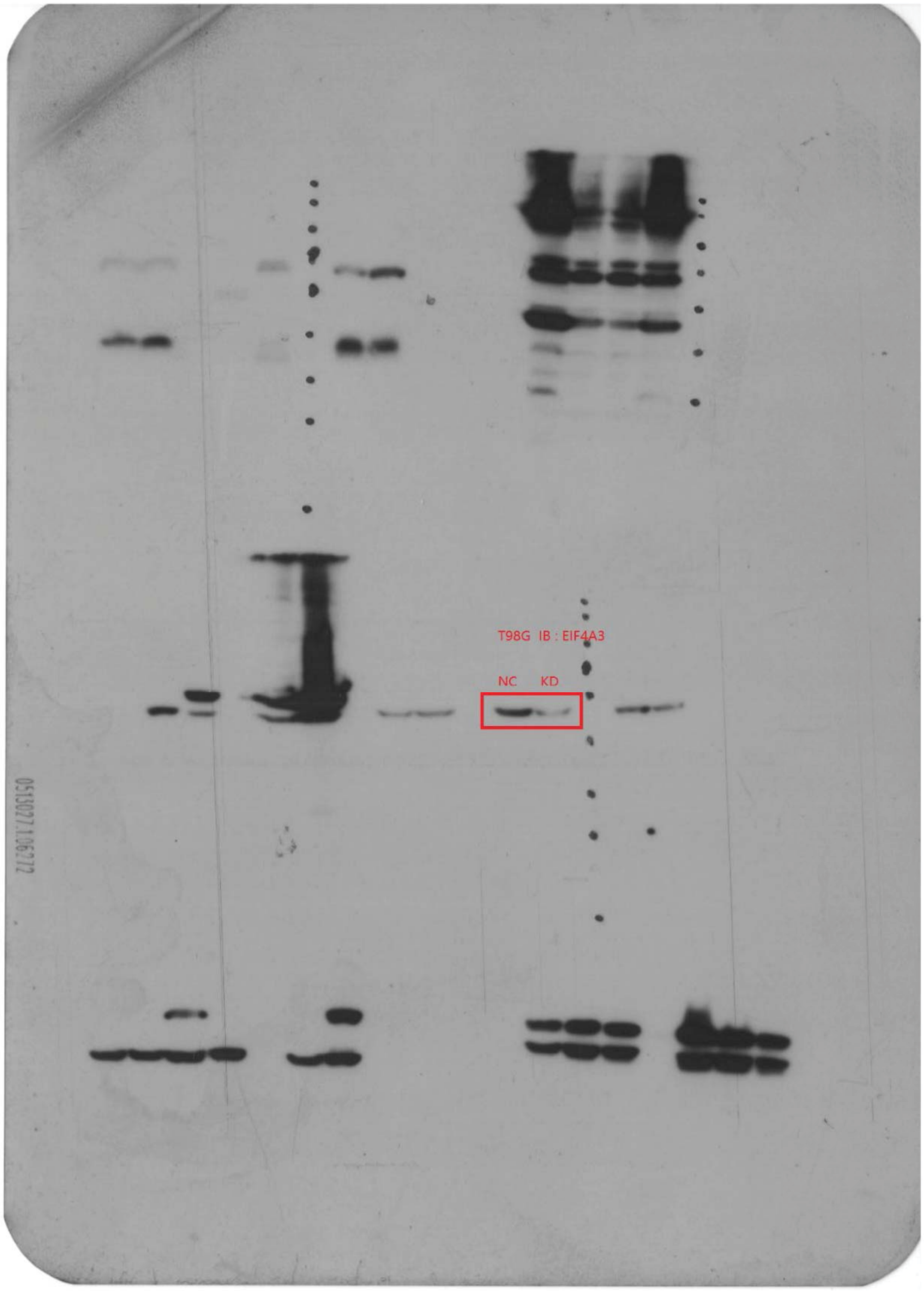

Figure 2a-U87, IB: Actin

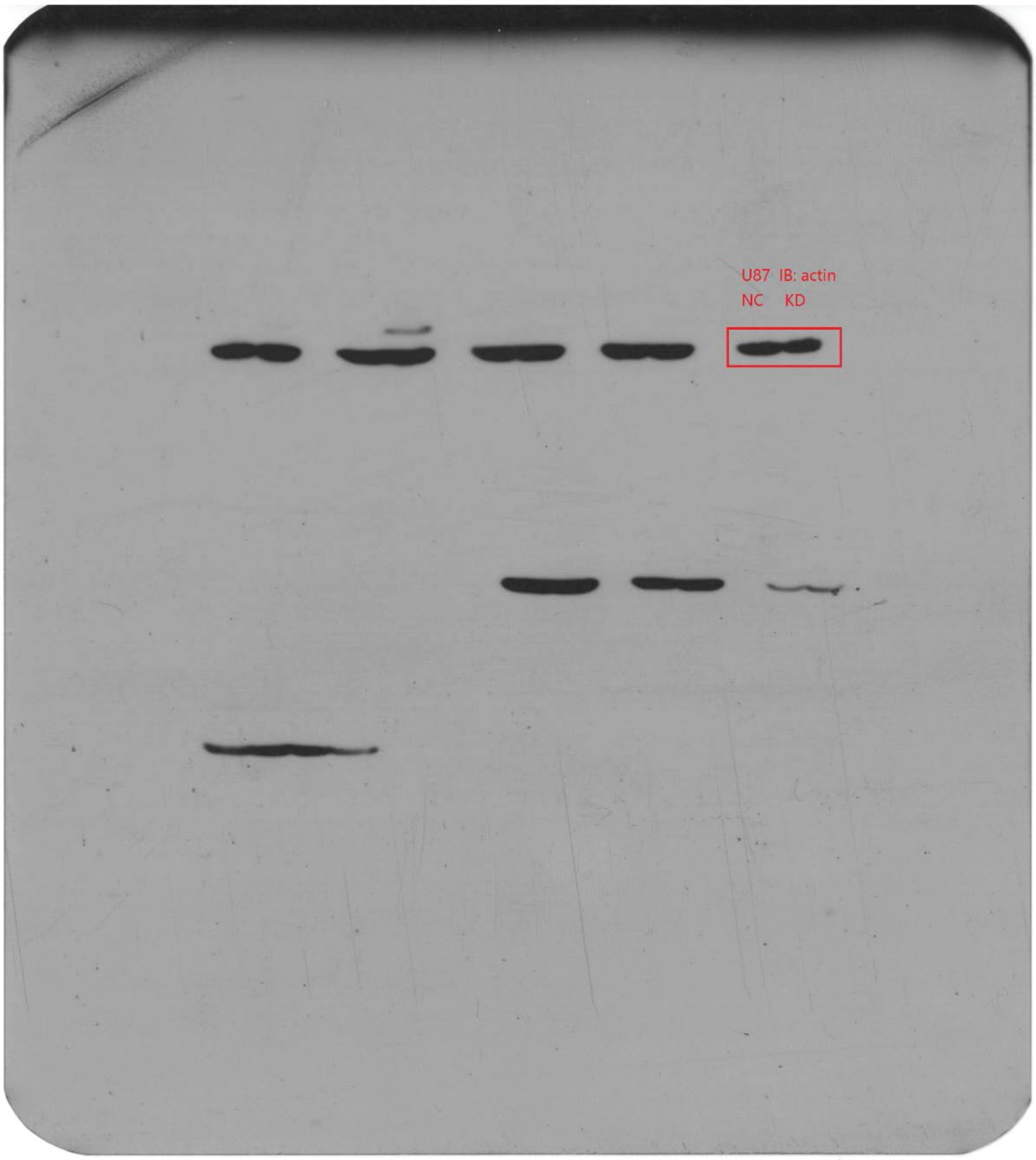

Figure 2a-T98g, IB: Actin

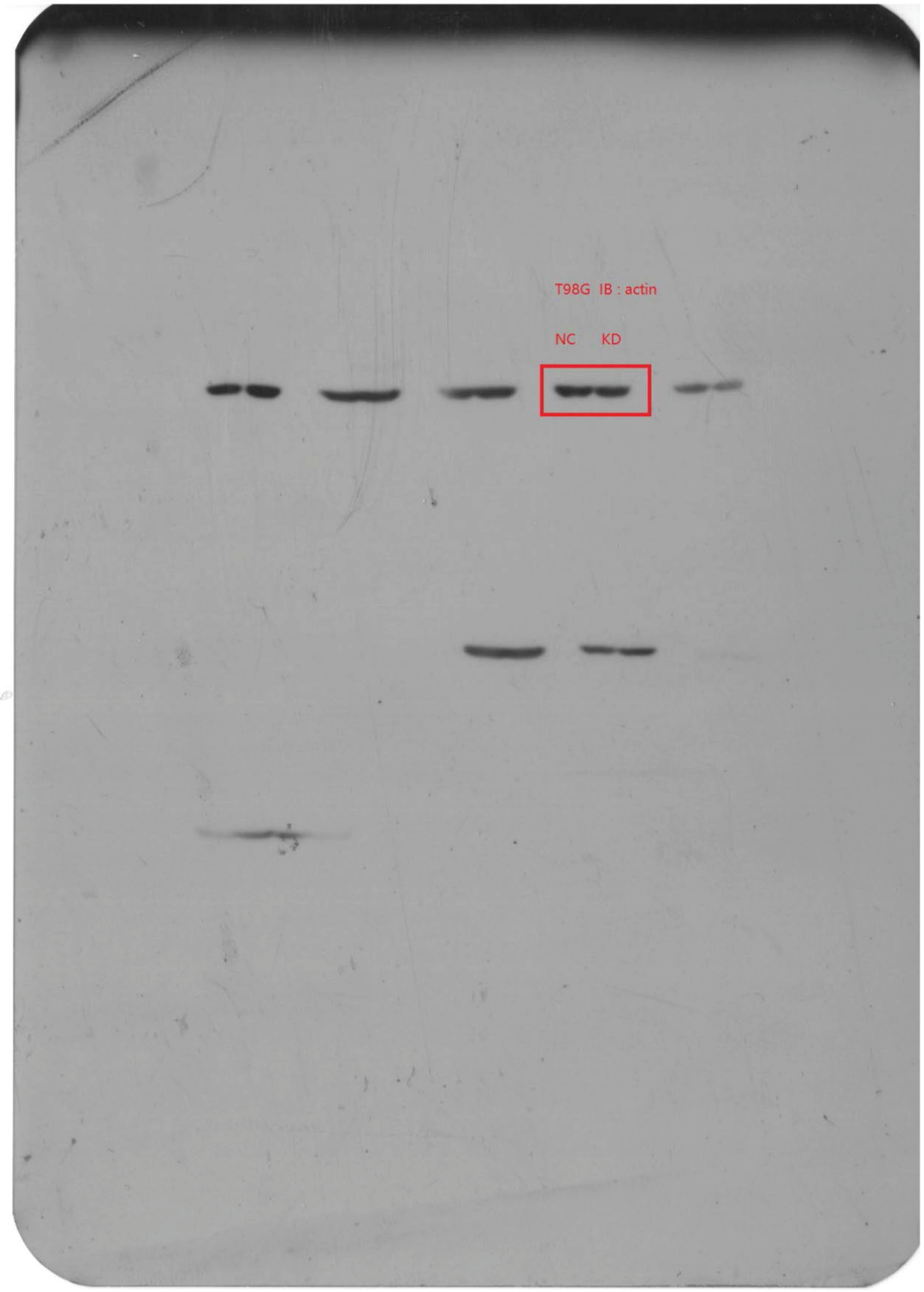

Figure 4a-A172-p-STAT3

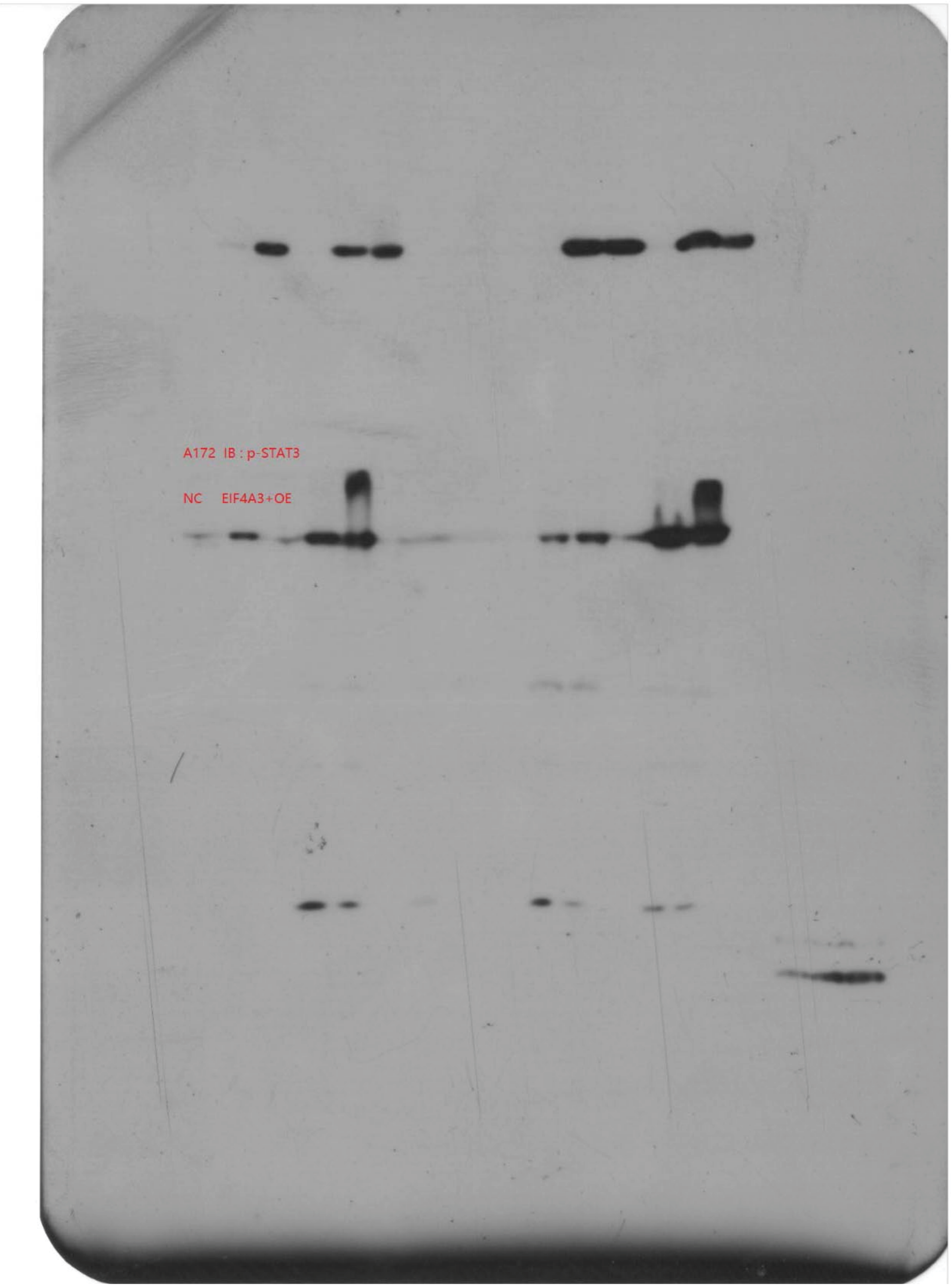

Figure 4a-U251-p-STAT3

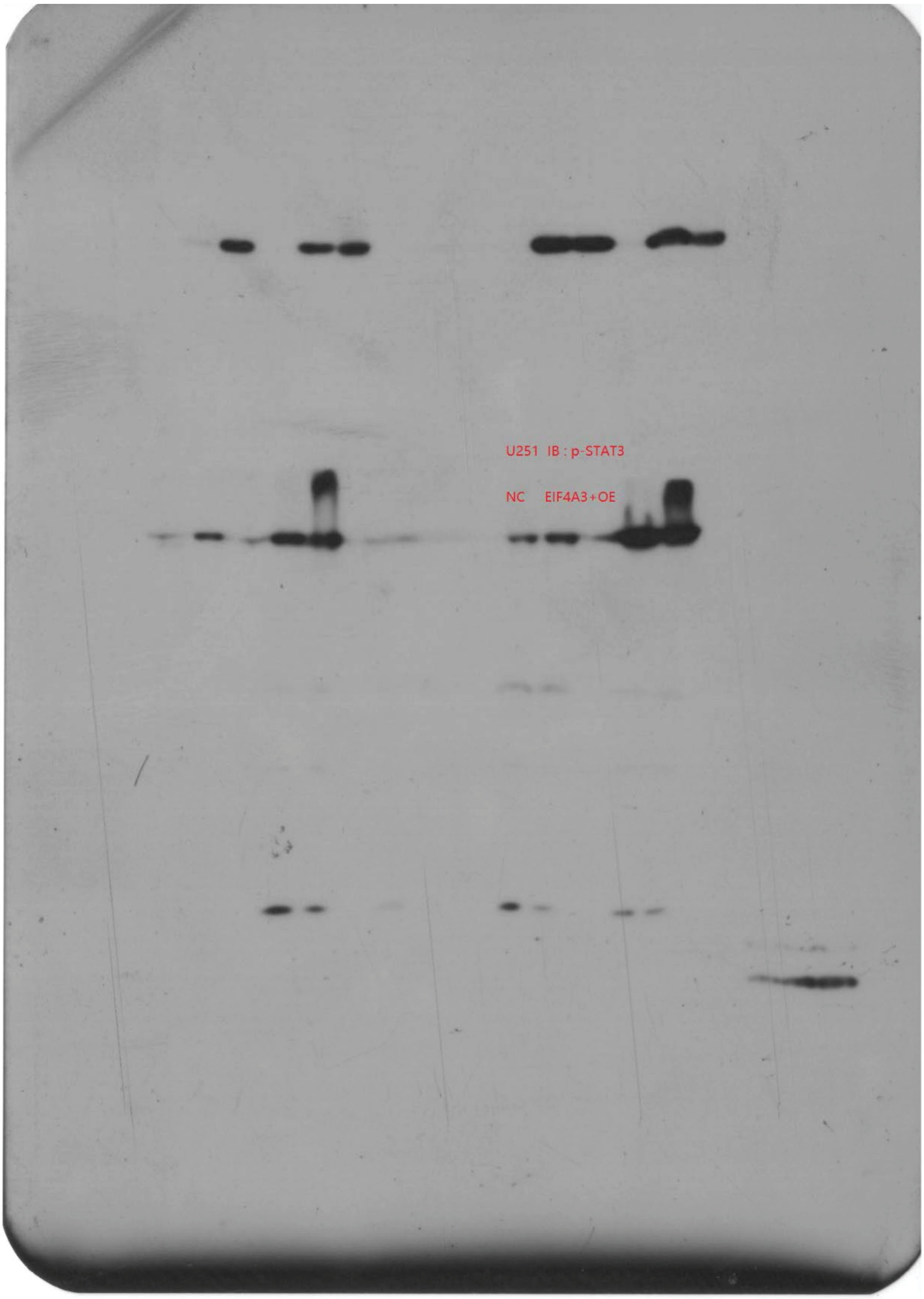

Figure 4a-U87-p-STAT3

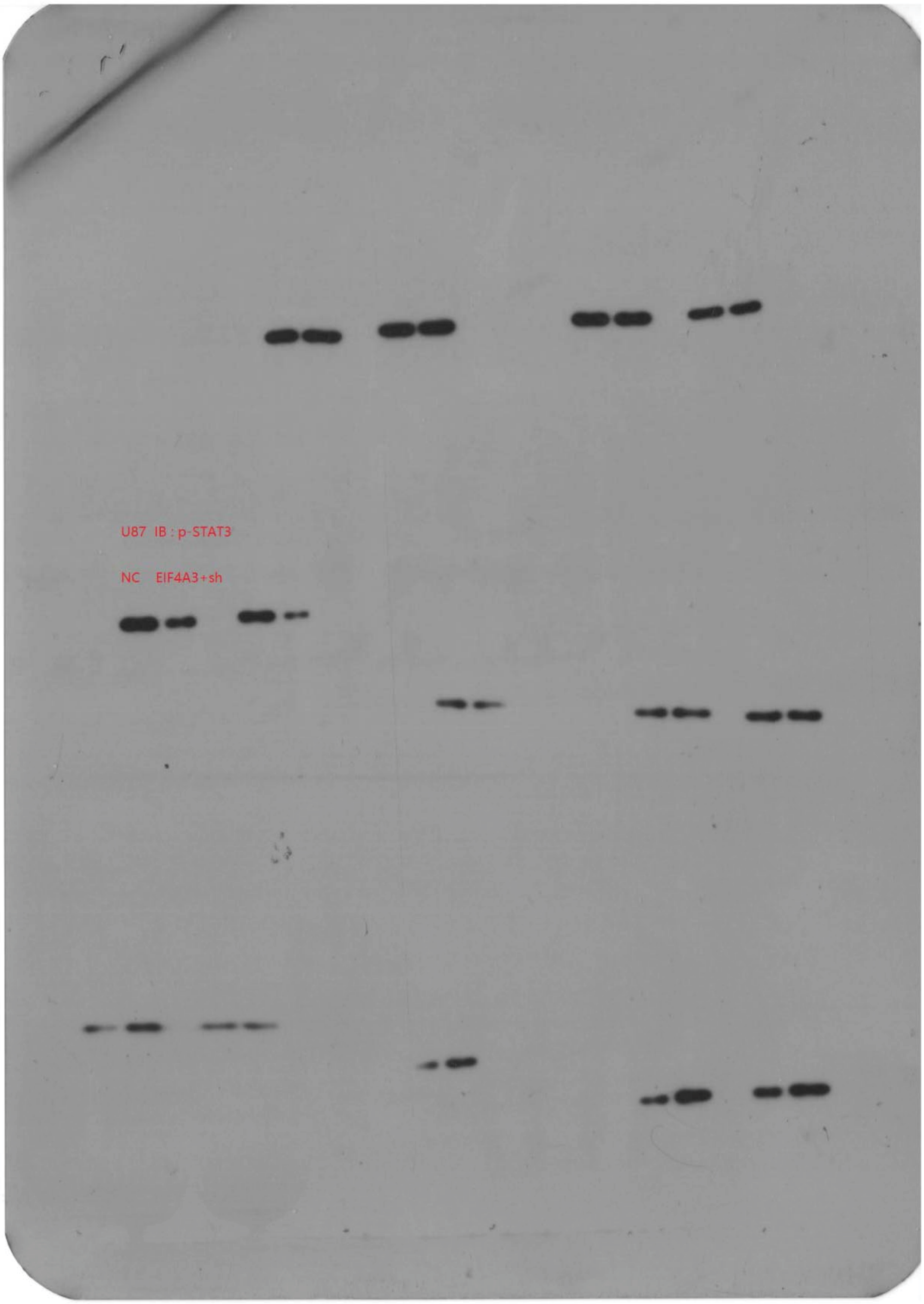

Figure 4a-T98G-p-STAT3

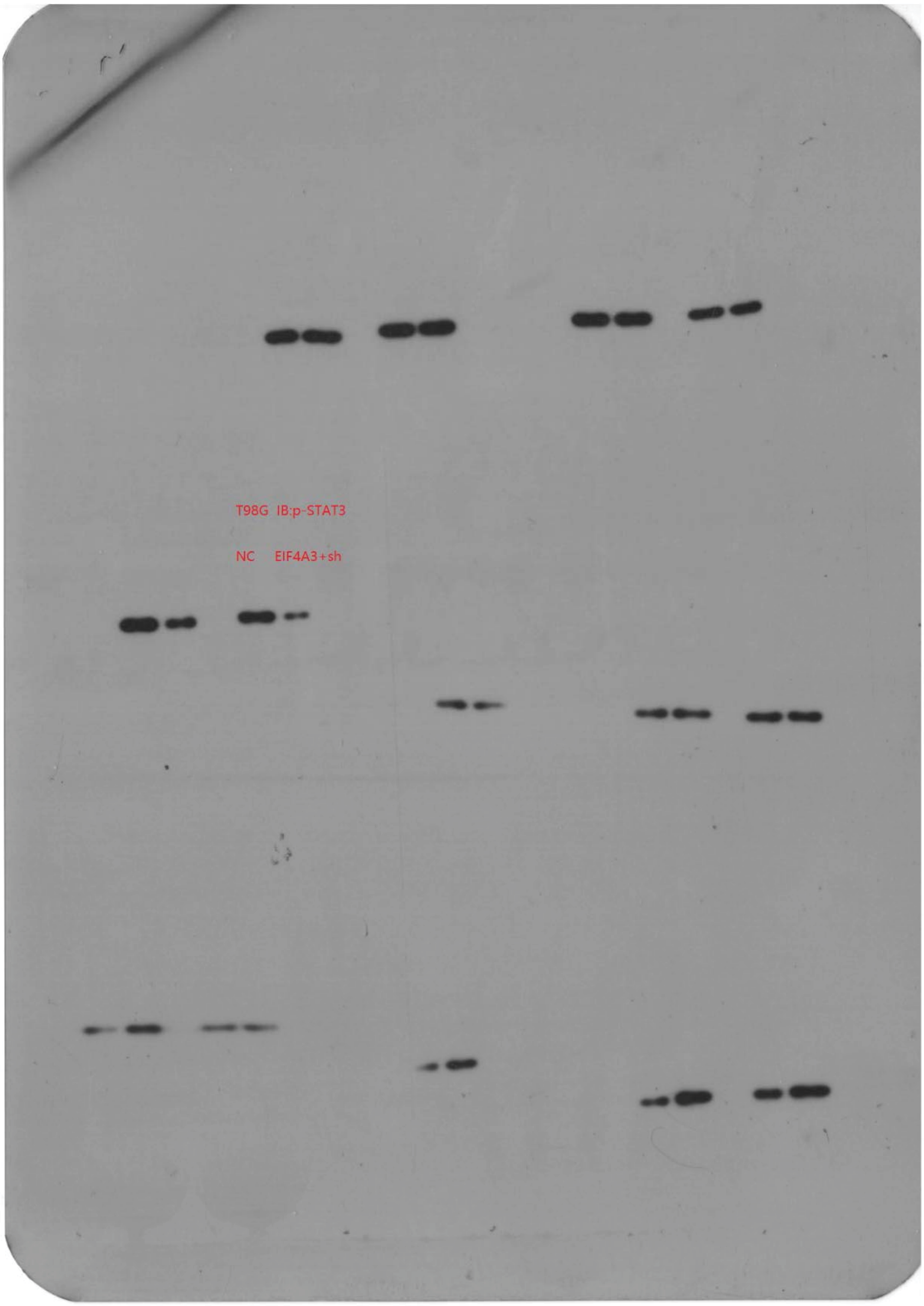

Figure 4a-A172-STAT3

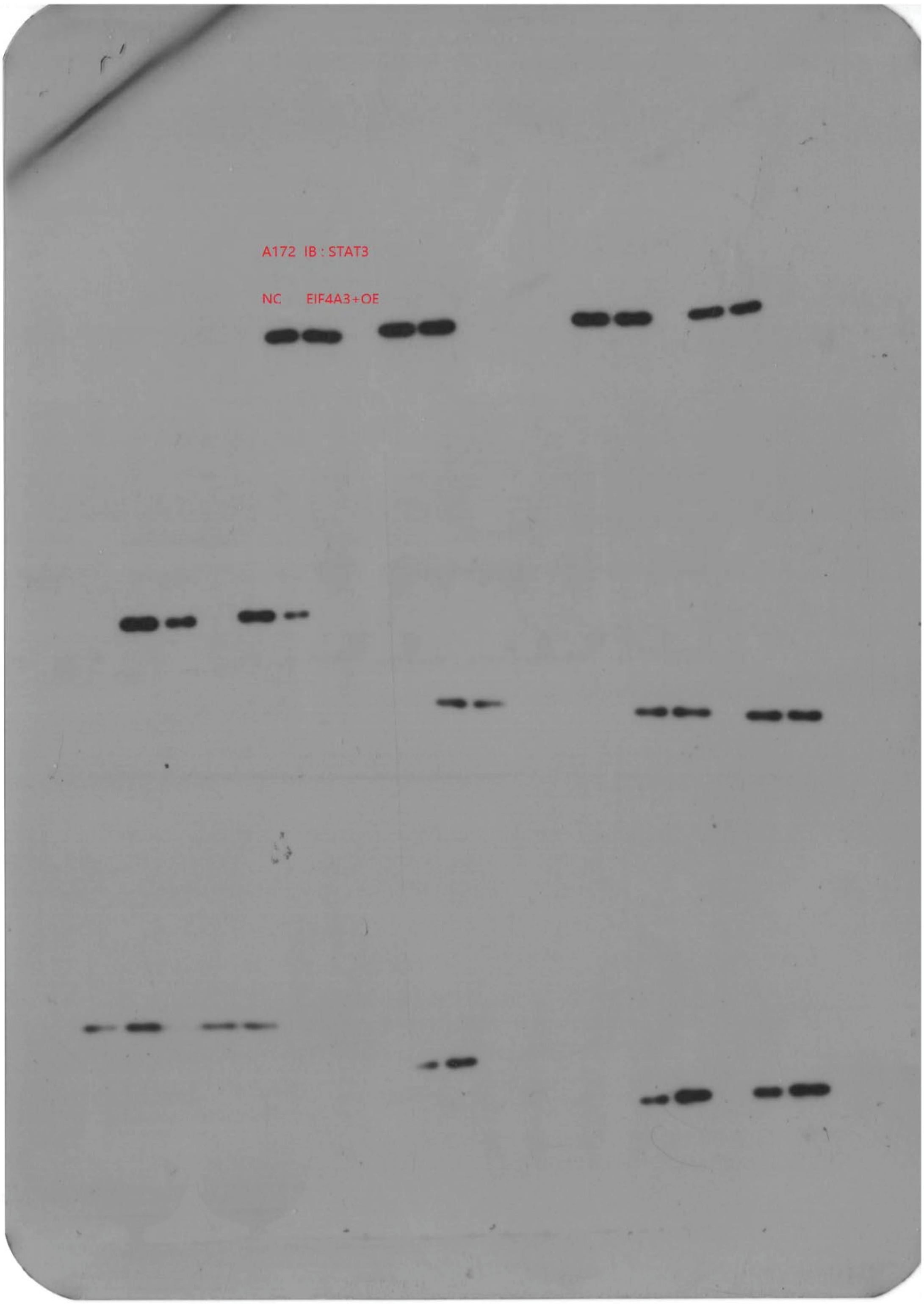

Figure 4a-U251-STAT3

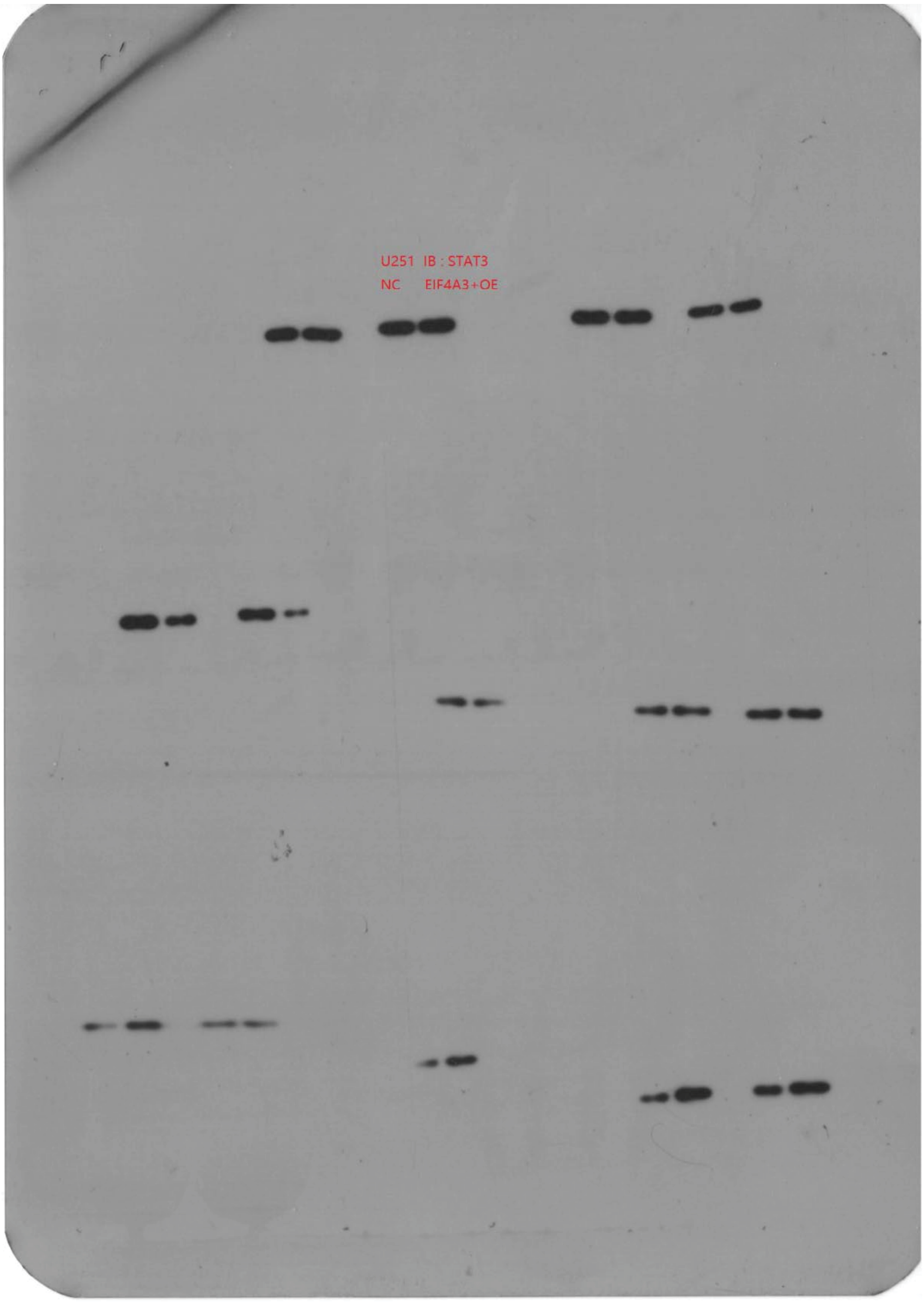

Figure 4a-U87-STAT3

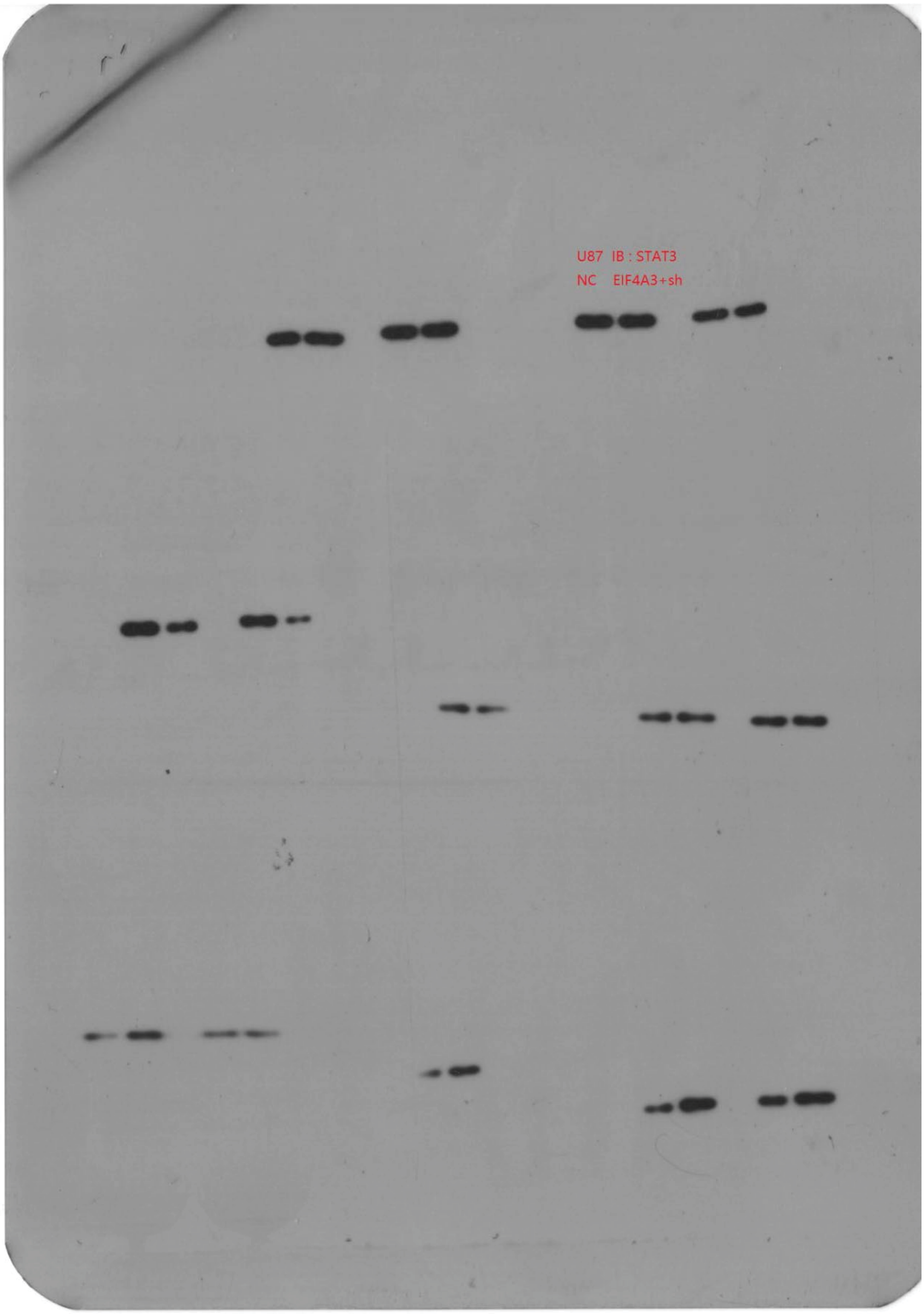

Figure 4a-T98G-STAT3

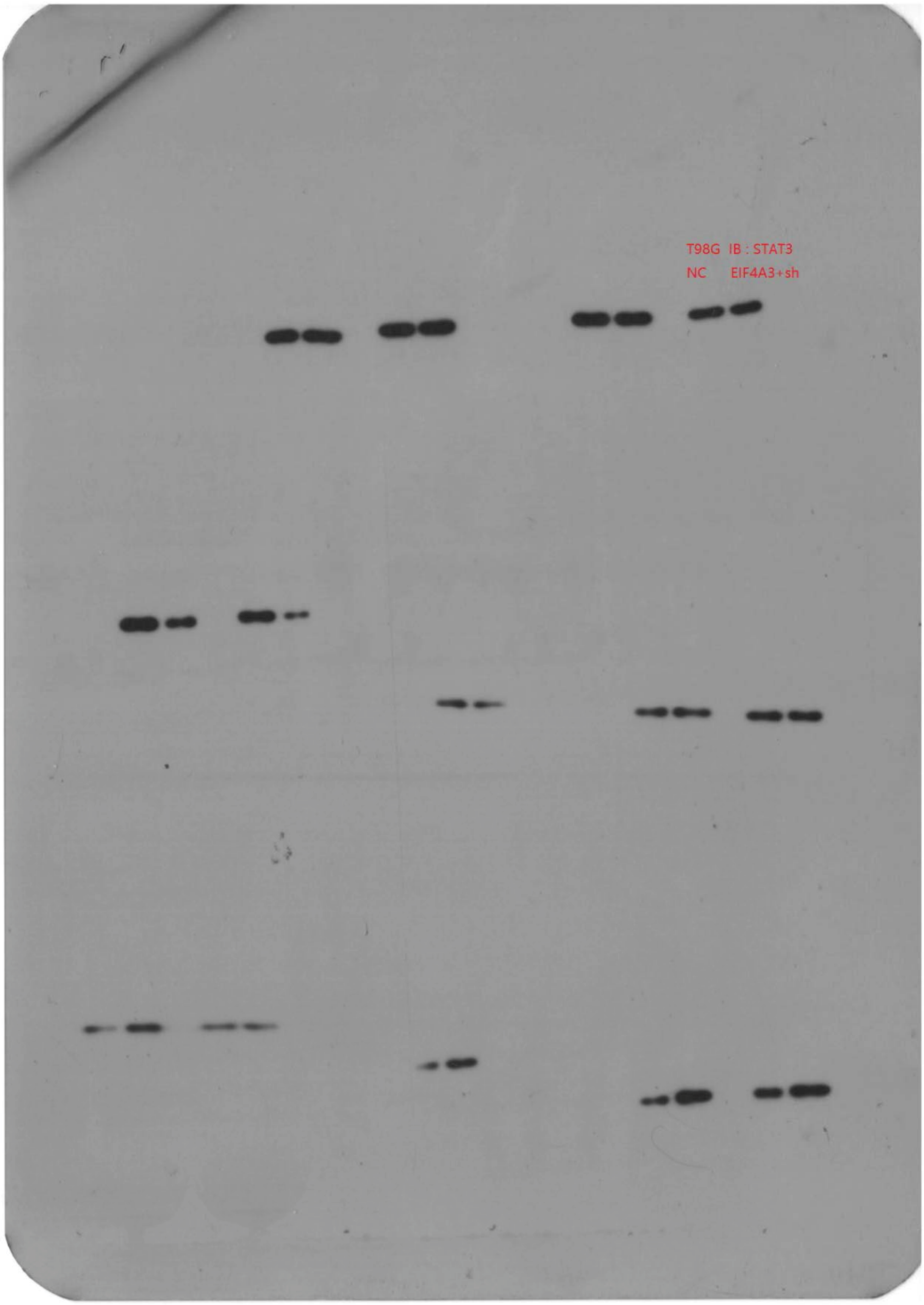

Figure 4a-A172-p-H3

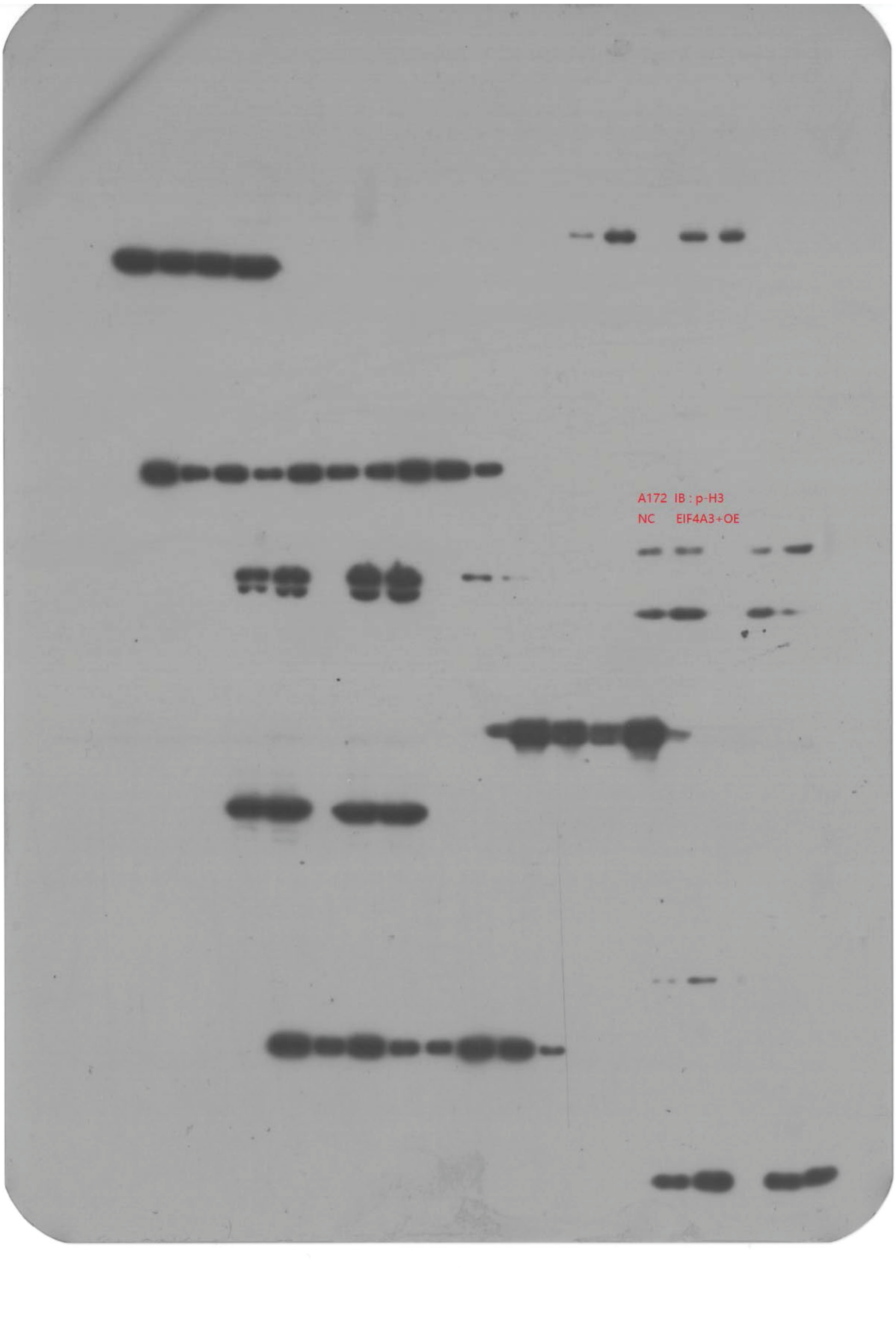

Figure 4a-U251-p-H3

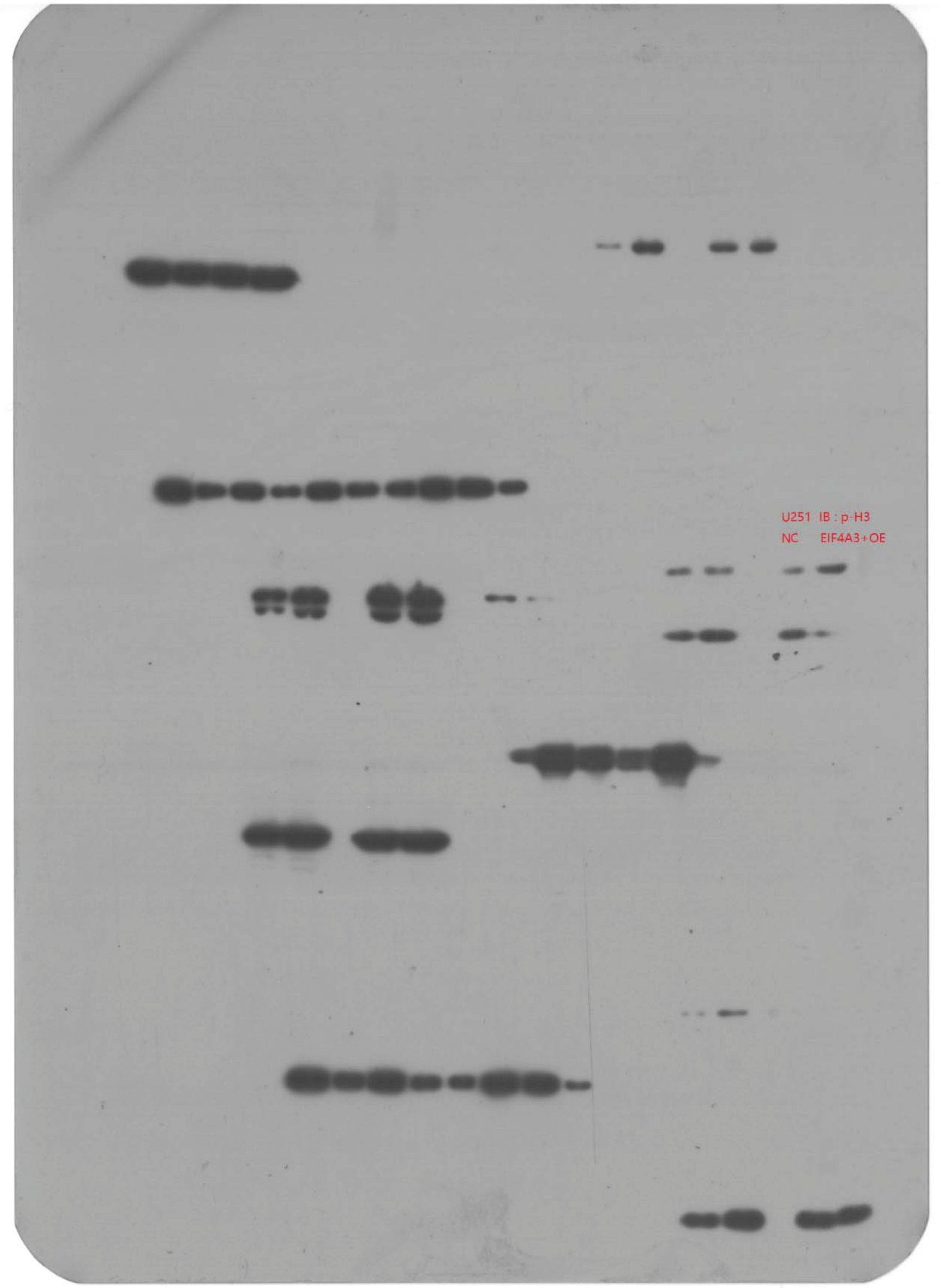

Figure 4a-U87-p-H3

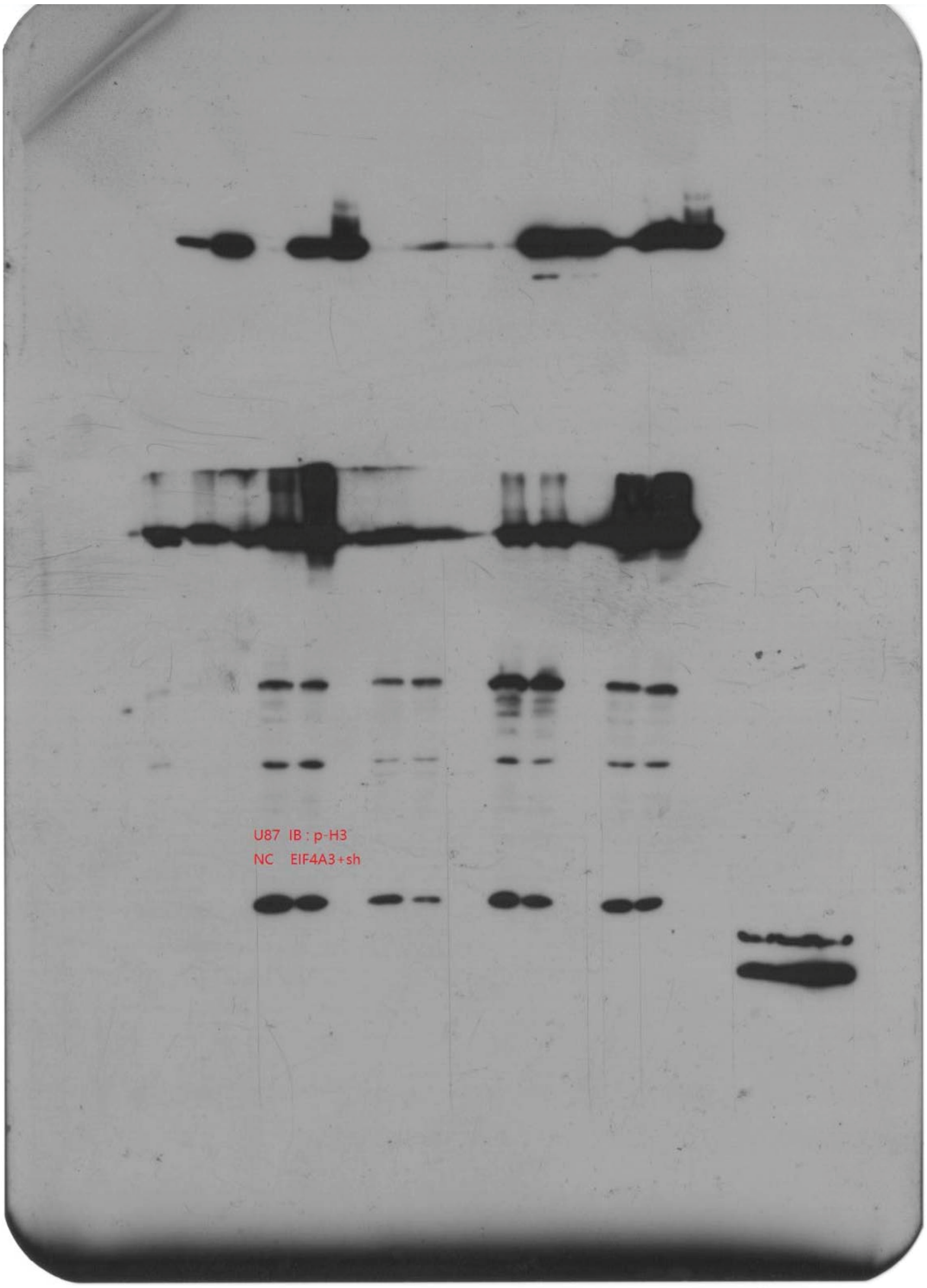

Figure 4a-T98G-p-H3

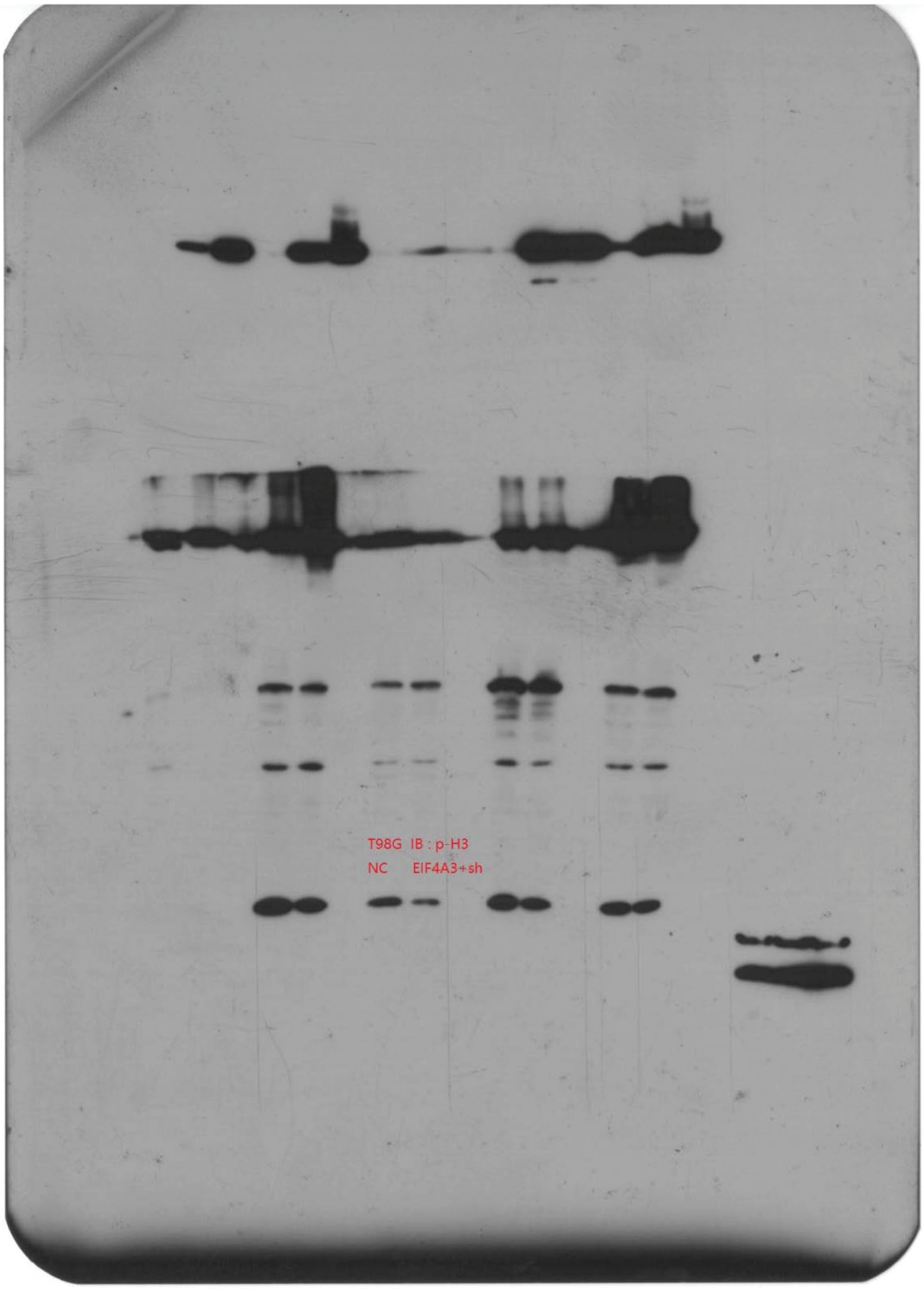

Figure 4a-A172-H3

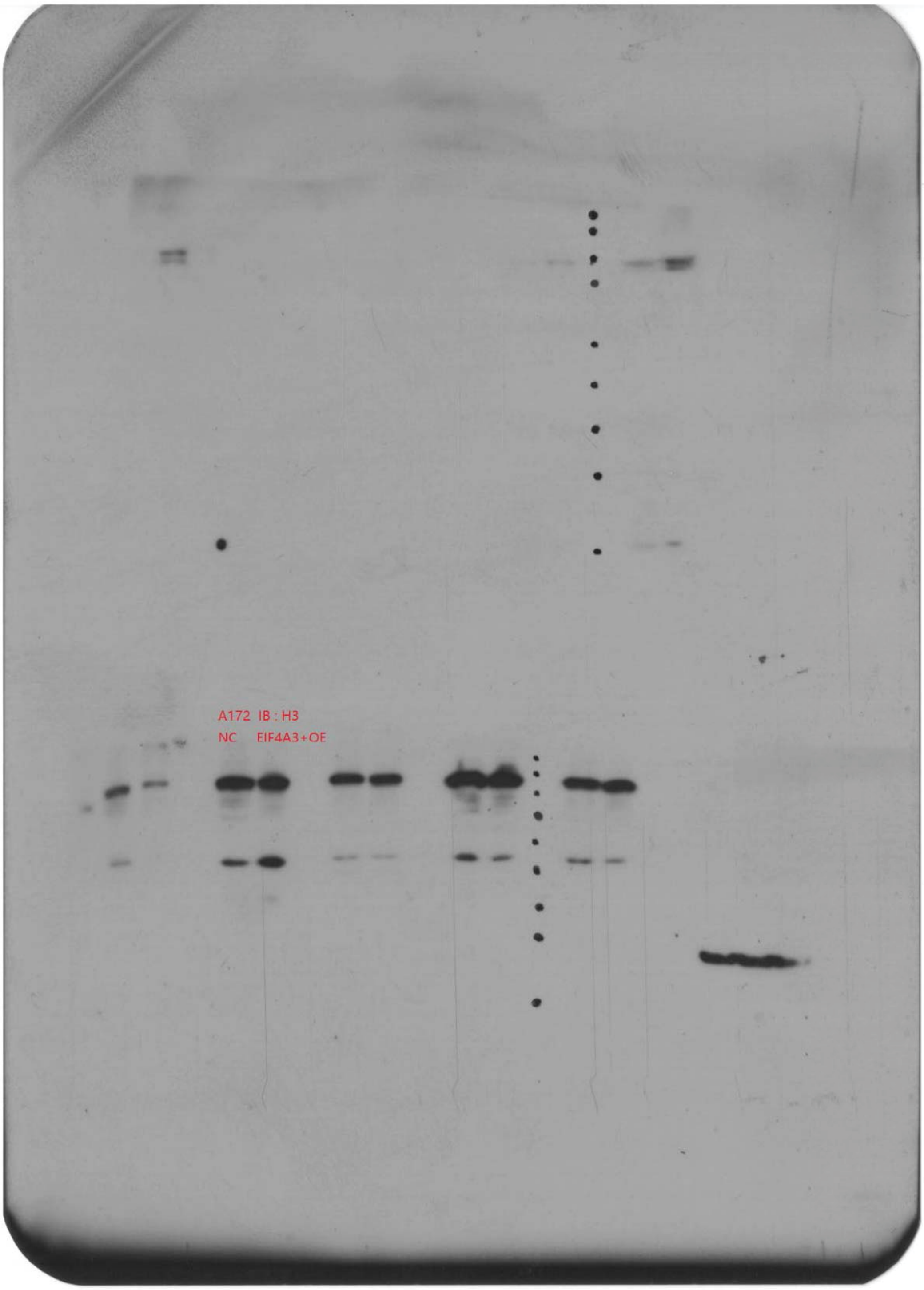

Figure 4a-U251-H3

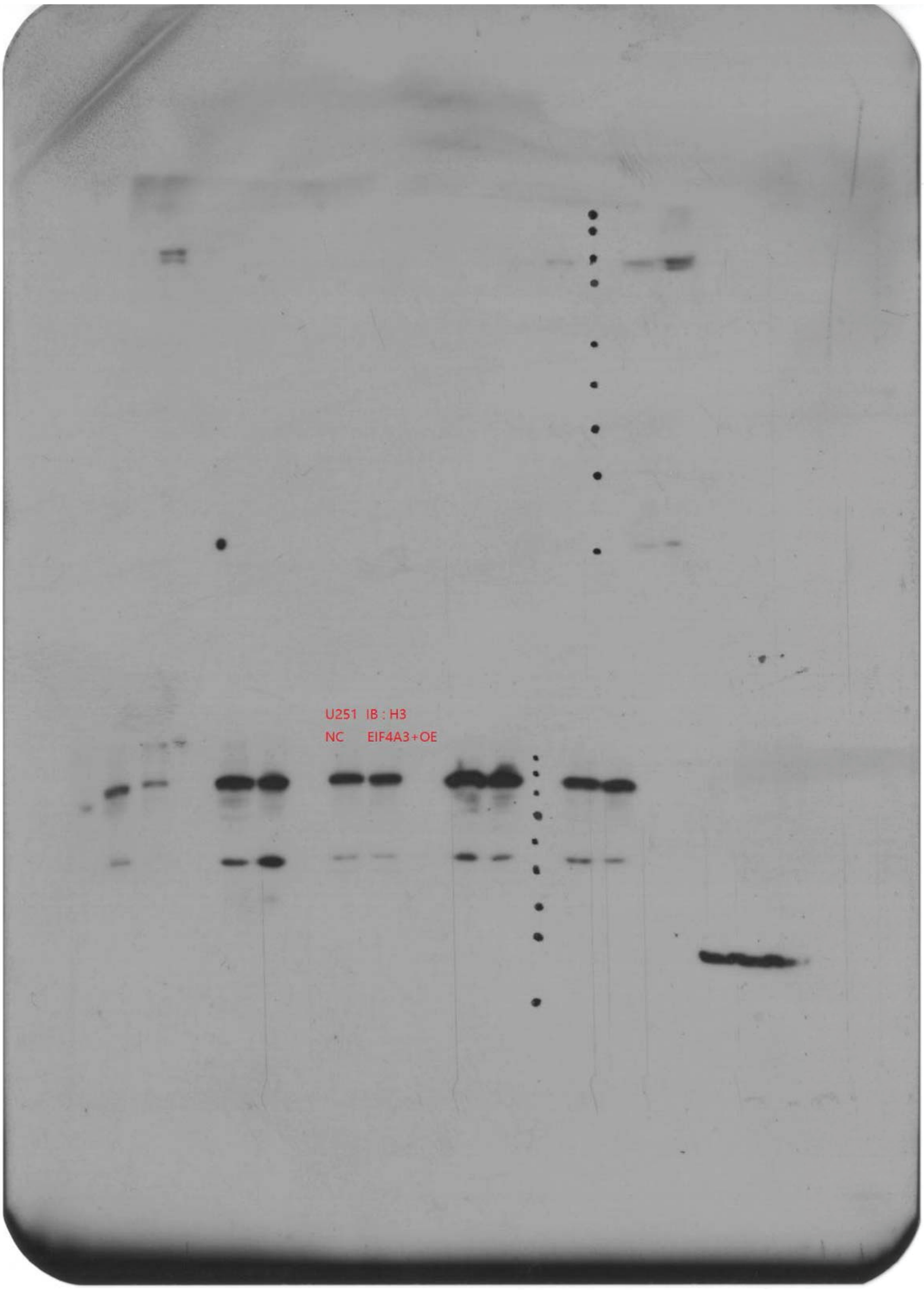

Figure 4a-U87-H3

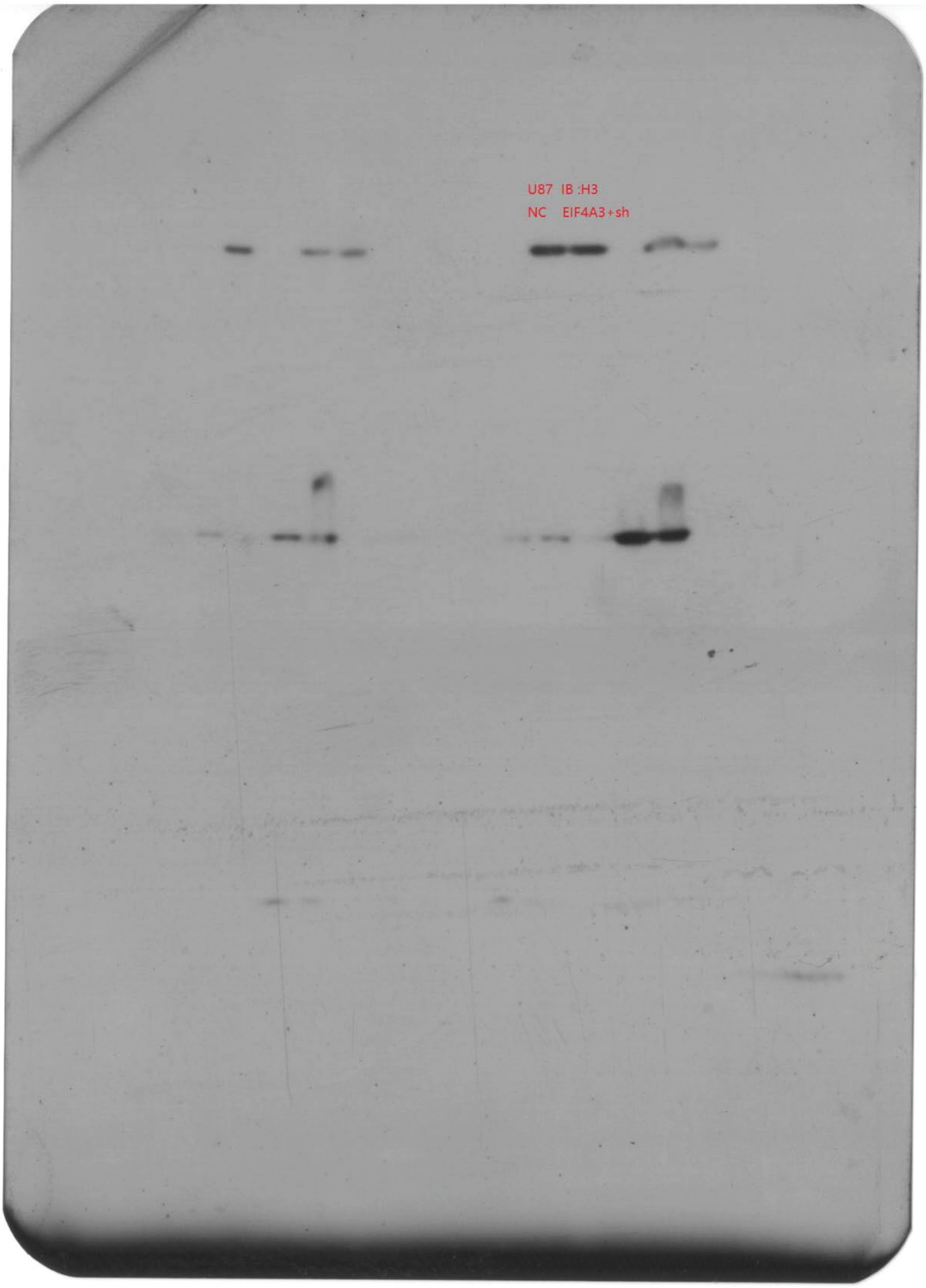

Figure 4a-T98G-H3

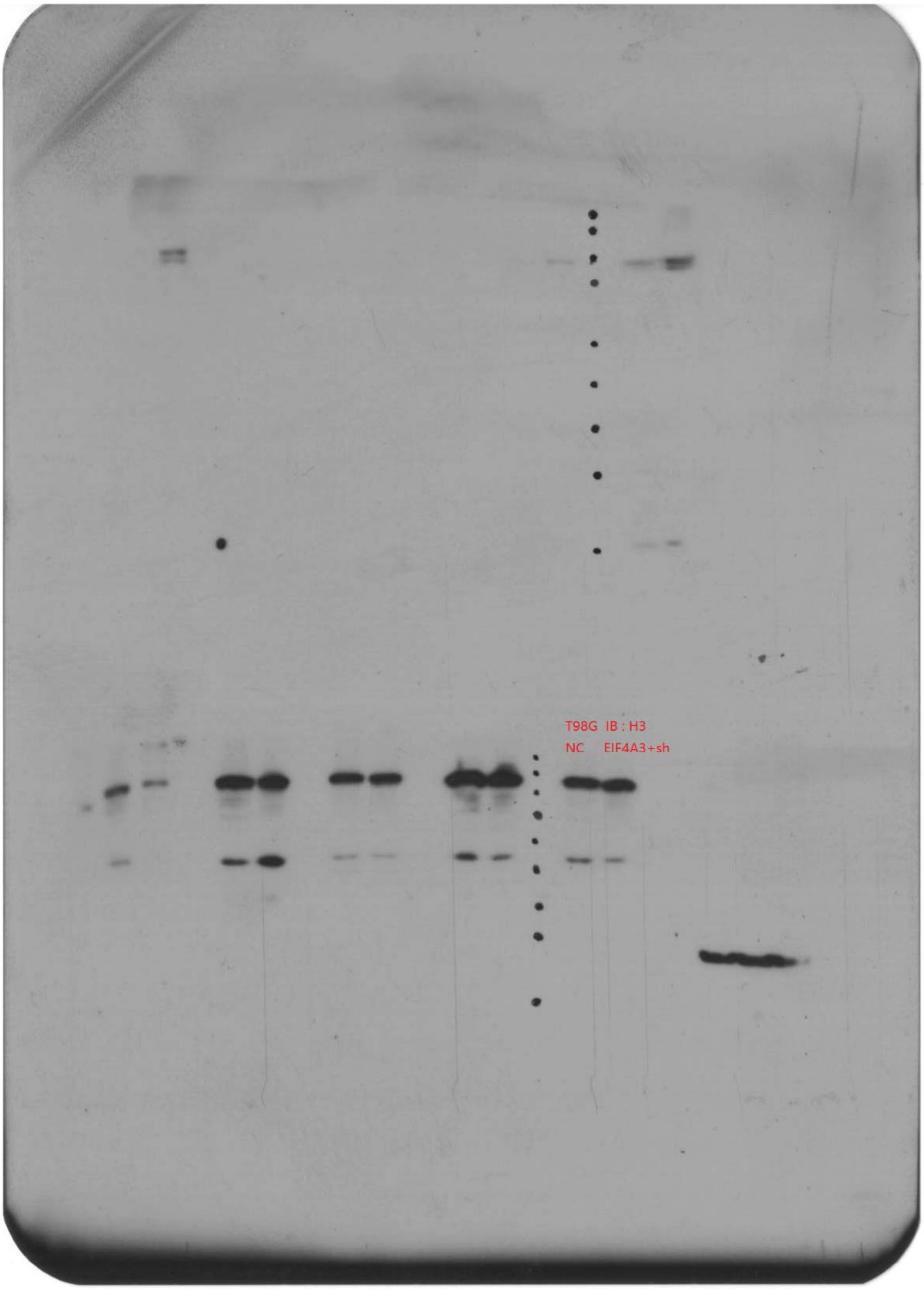

Figure 4a-A172-hes1

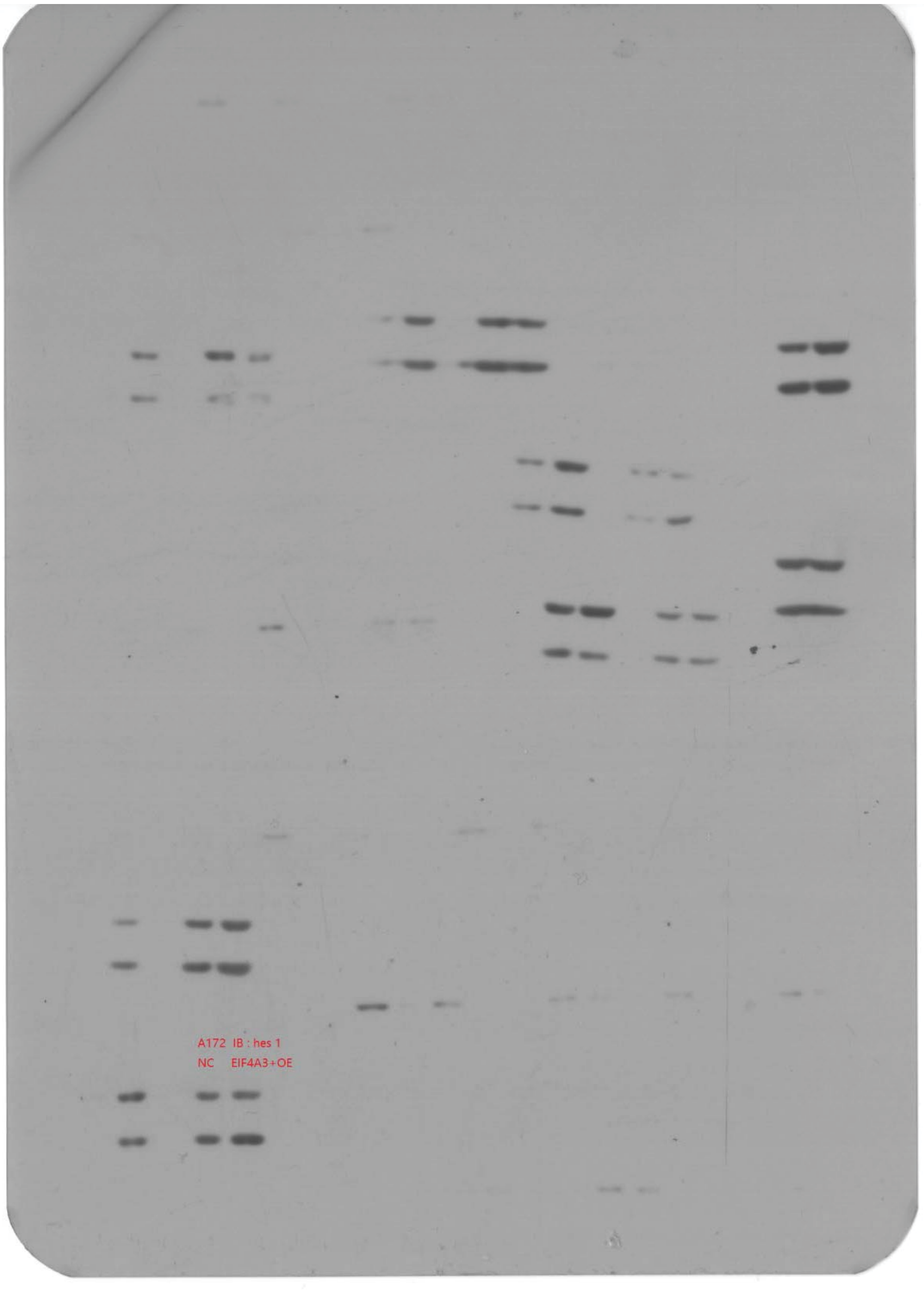

Figure 4a-U251-hes1

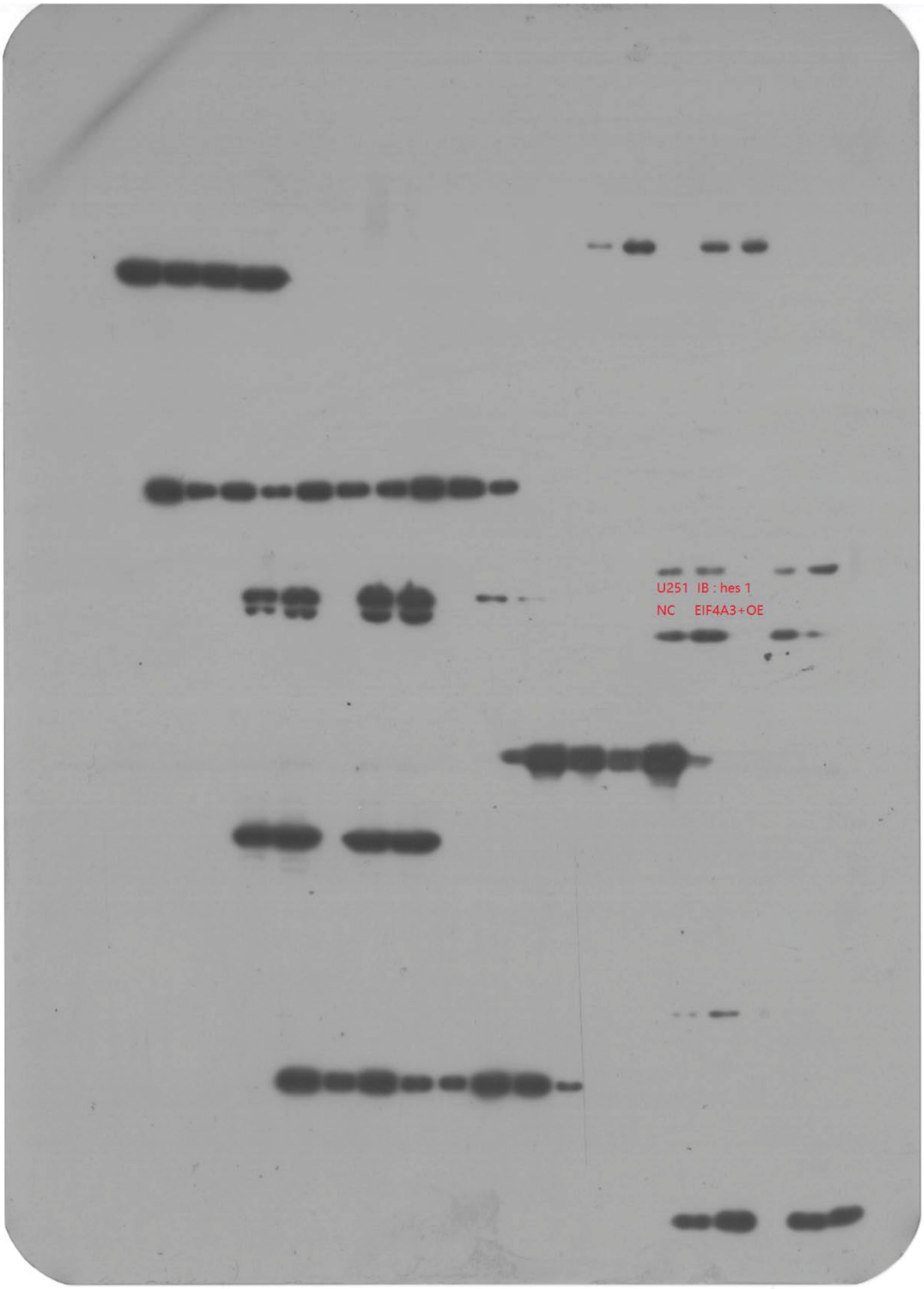

Figure 4a-U87-hes1

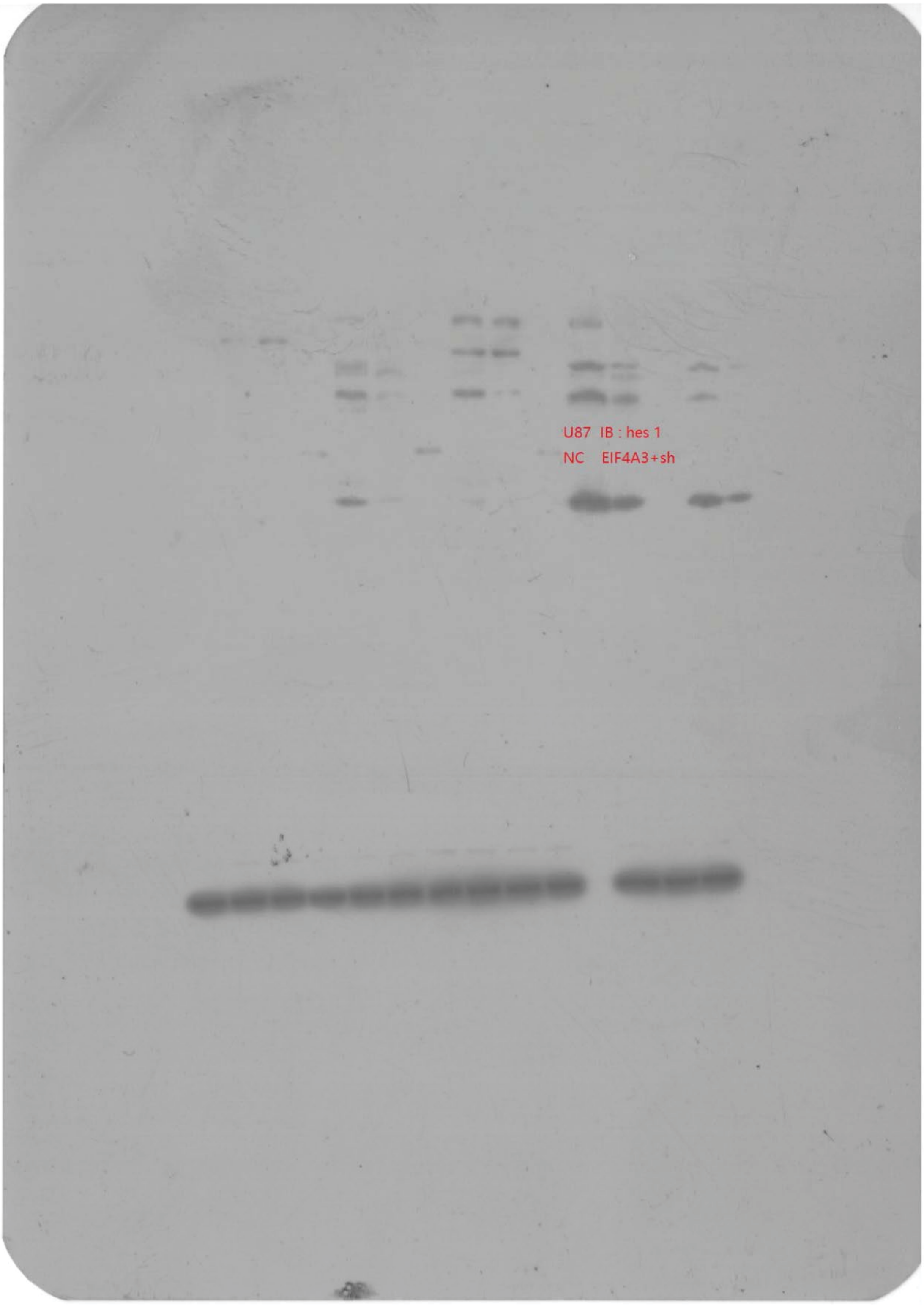

Figure 4a-T98G-hes1

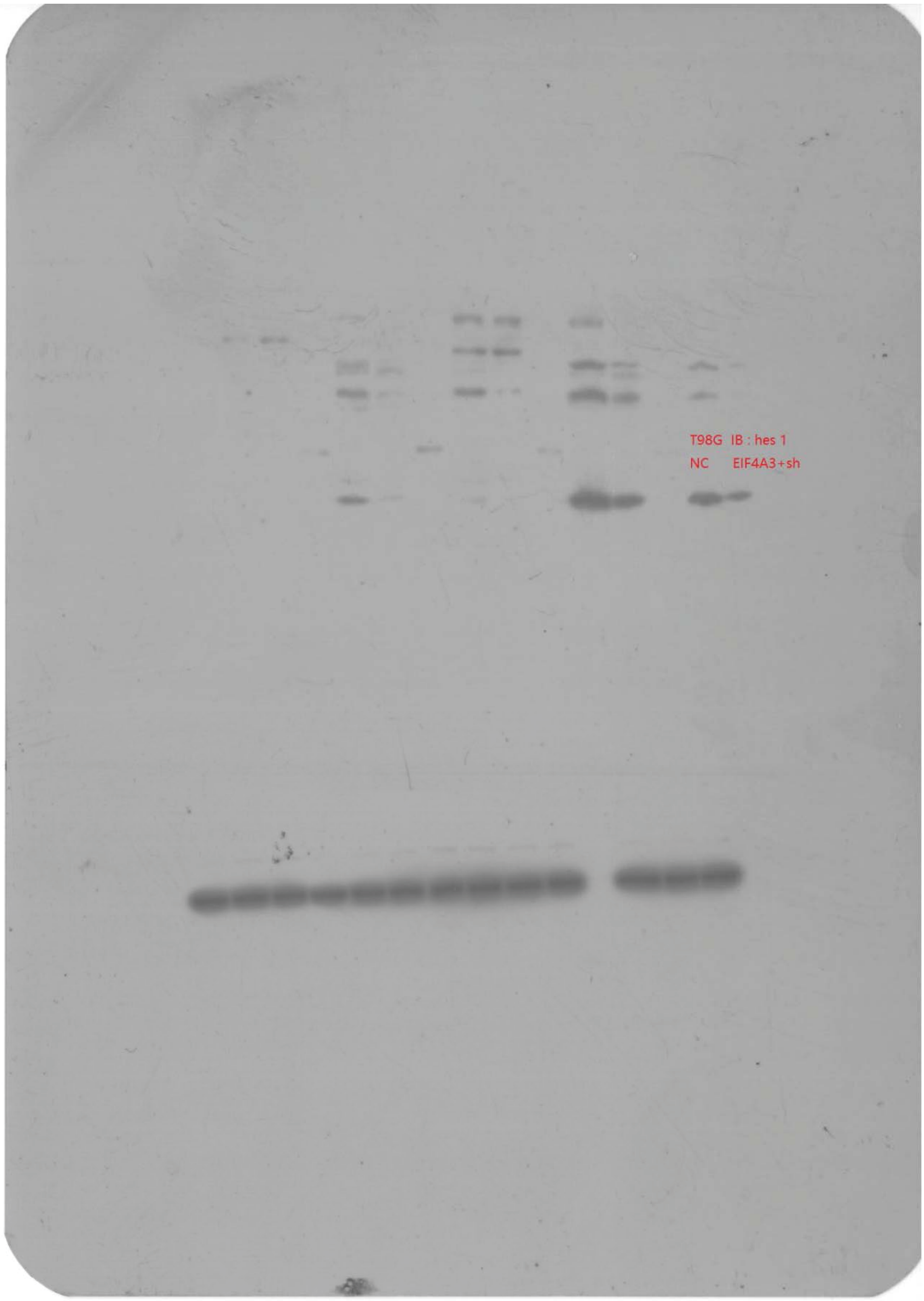

Figure 4a-A172-Notch1

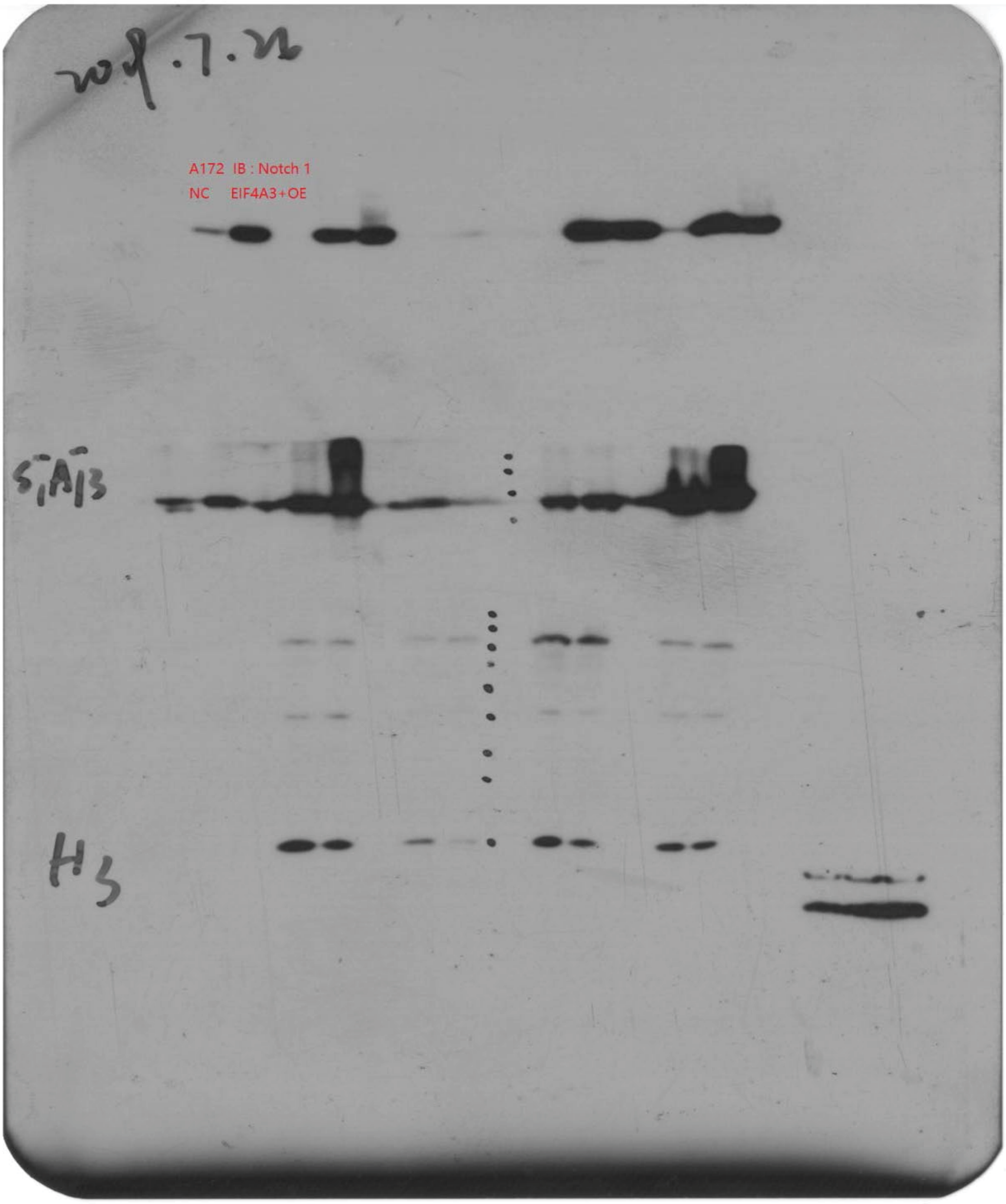

Figure 4a-U251-Notch1

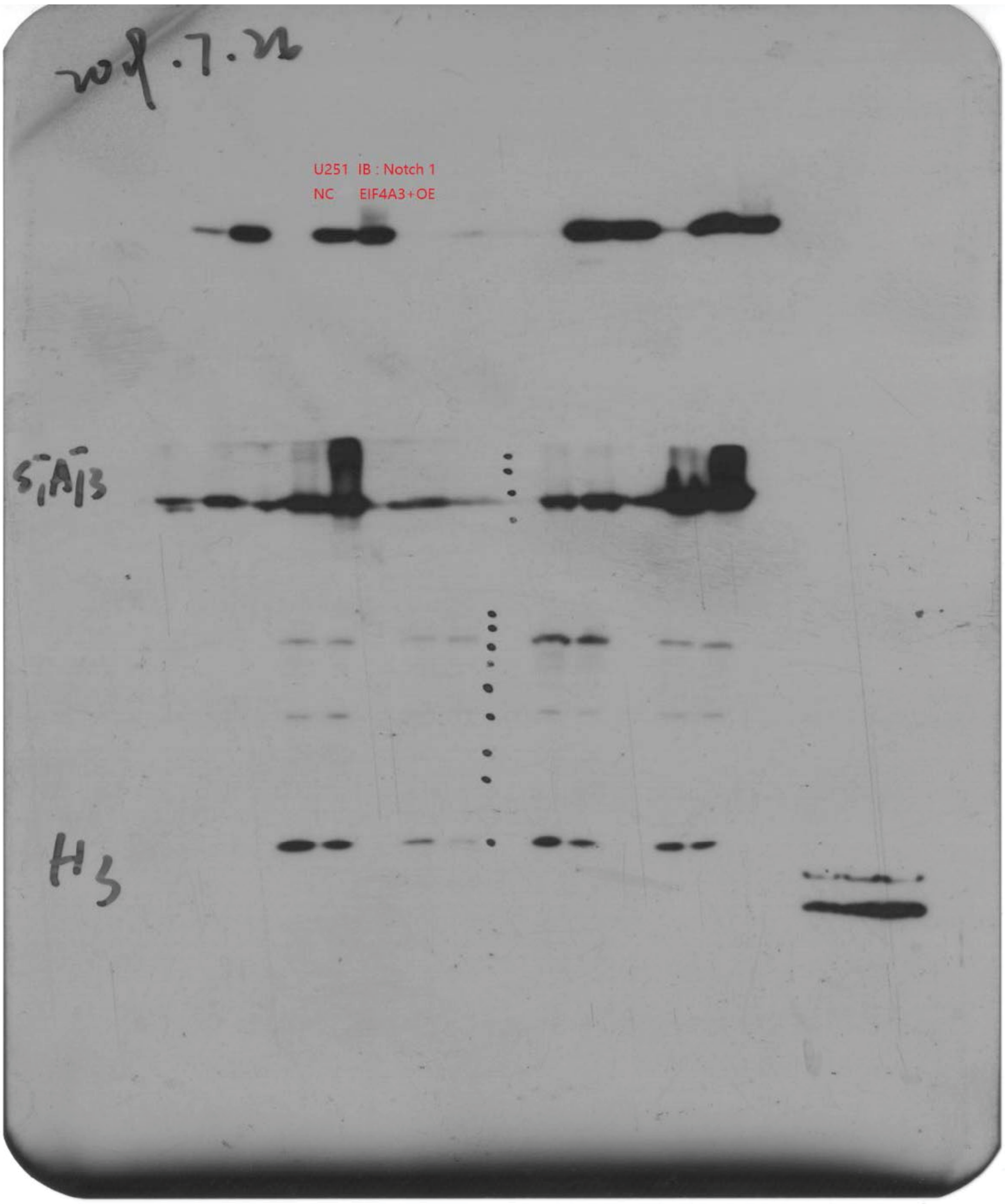

Figure 4a-U87-Notch1

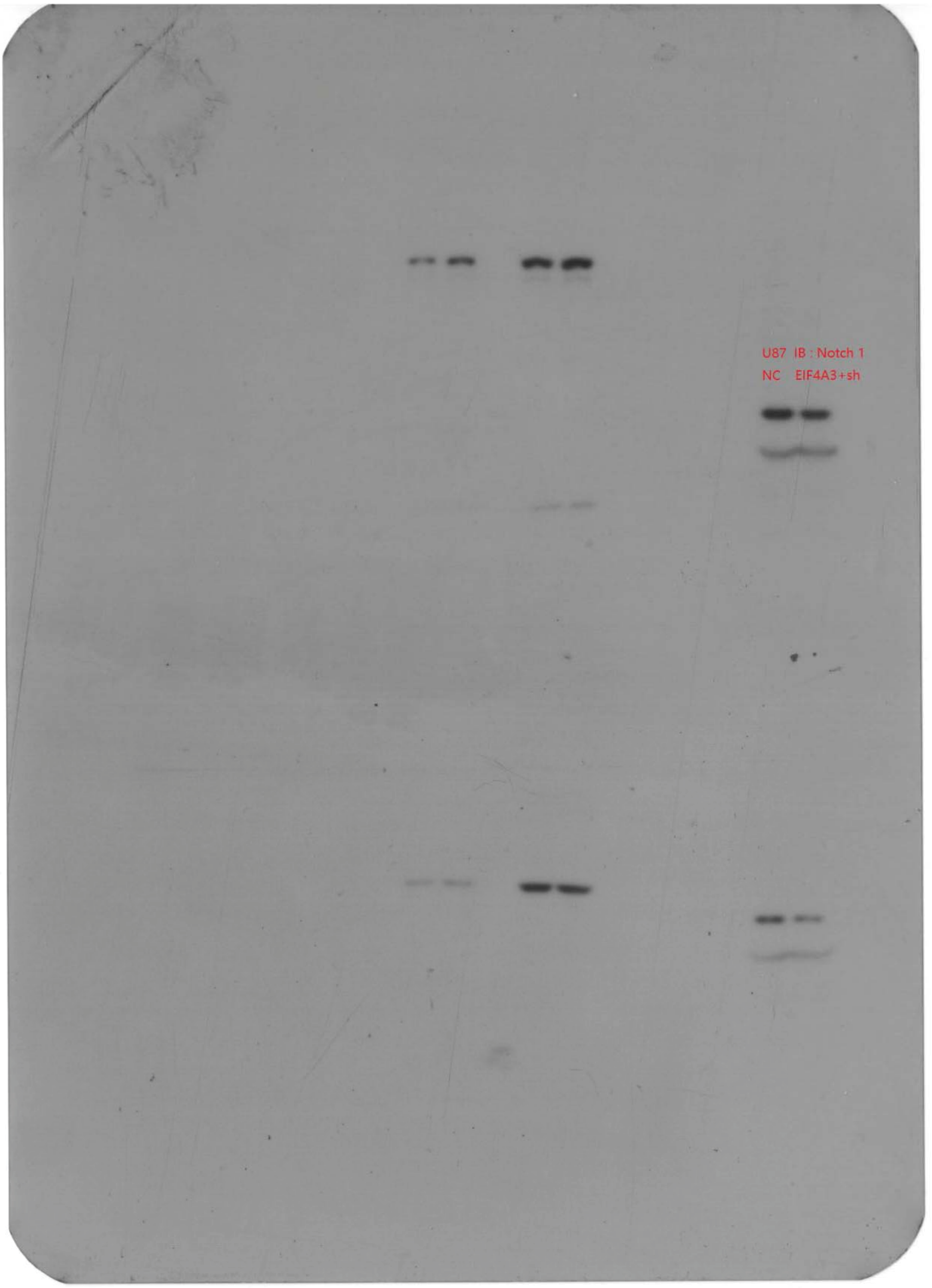

Figure 4a-T98G-Notch1

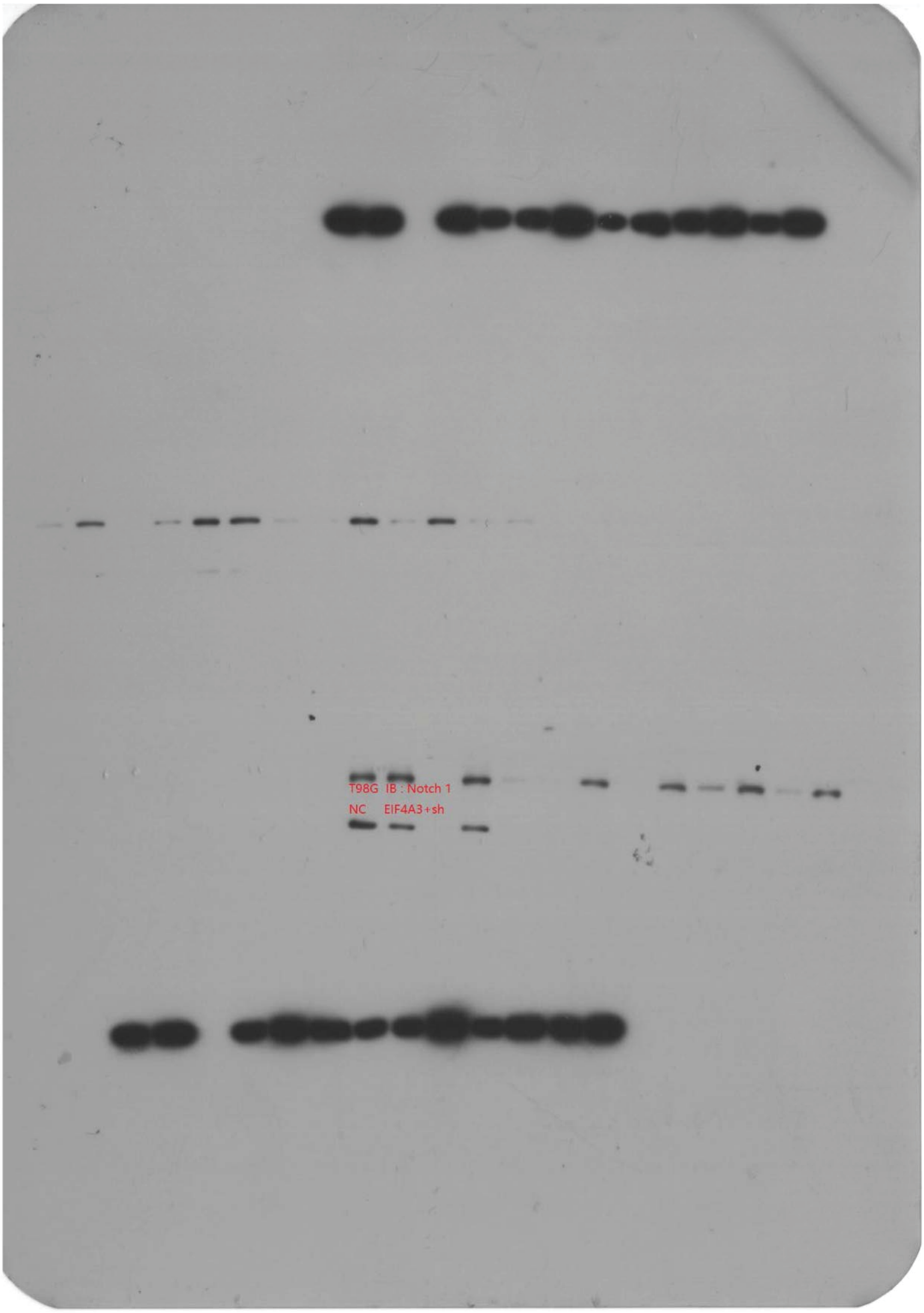

Figure 4a-A172-actin

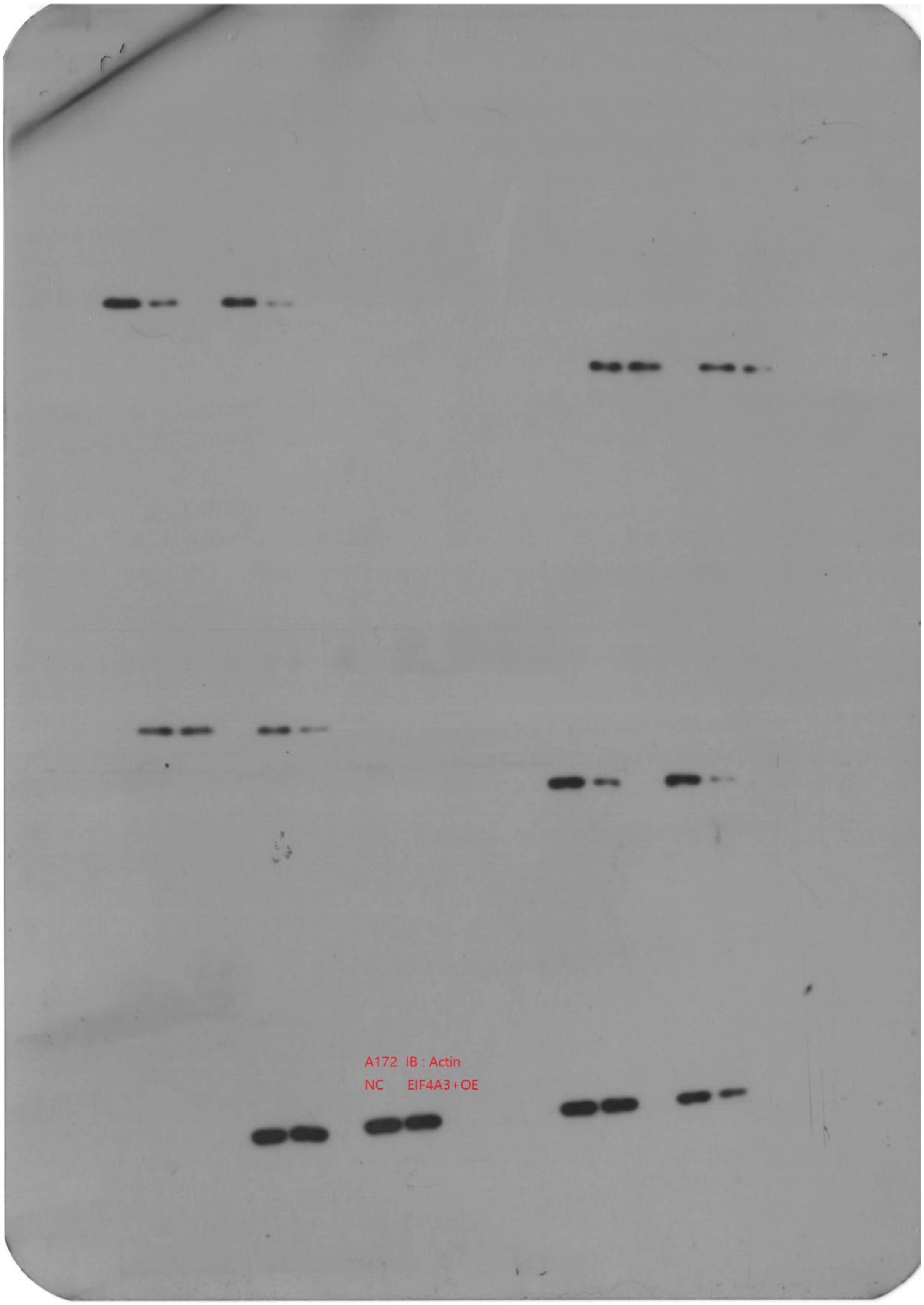

Figure 4a-U251-actin

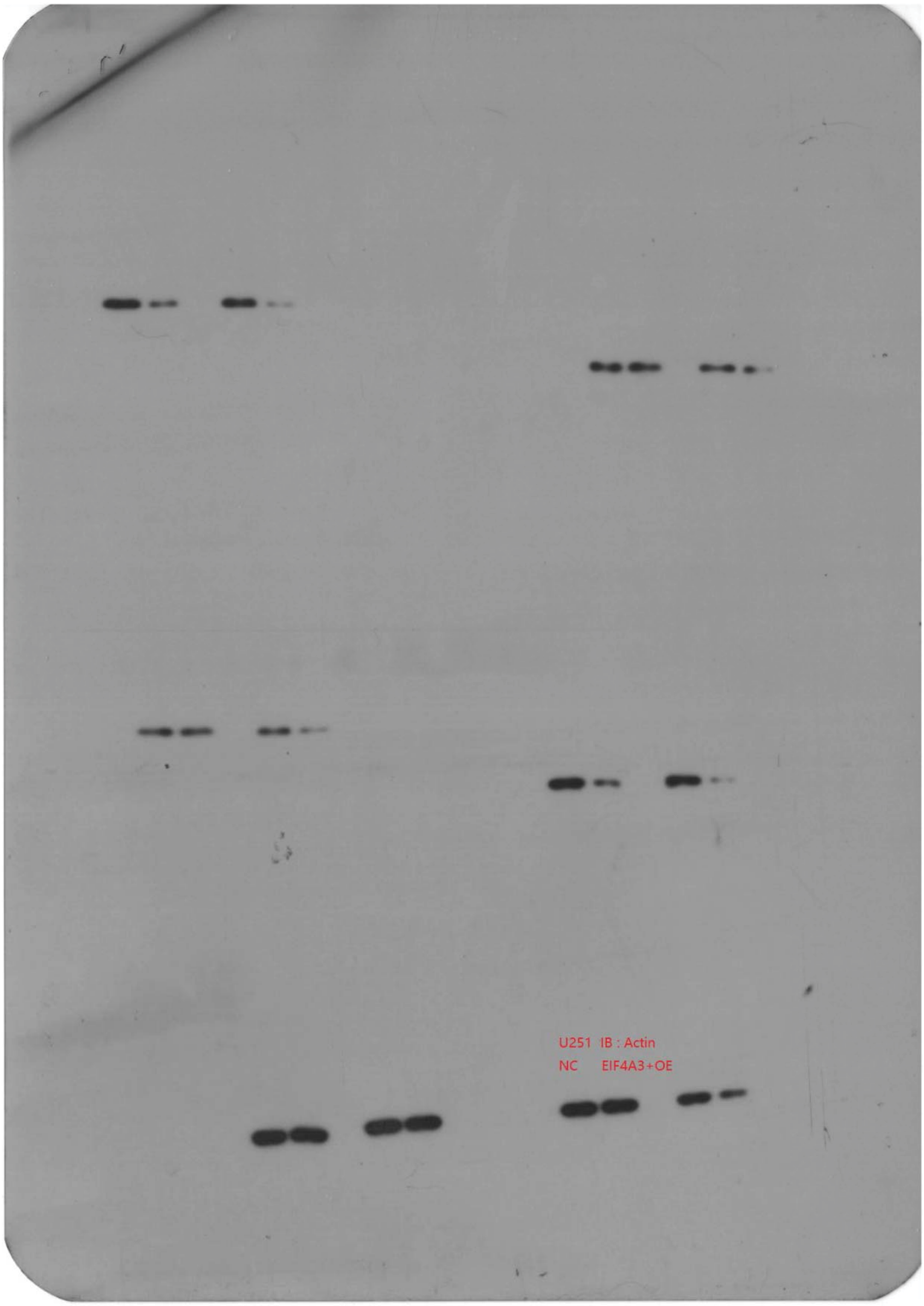

Figure 4a-U87-Actin

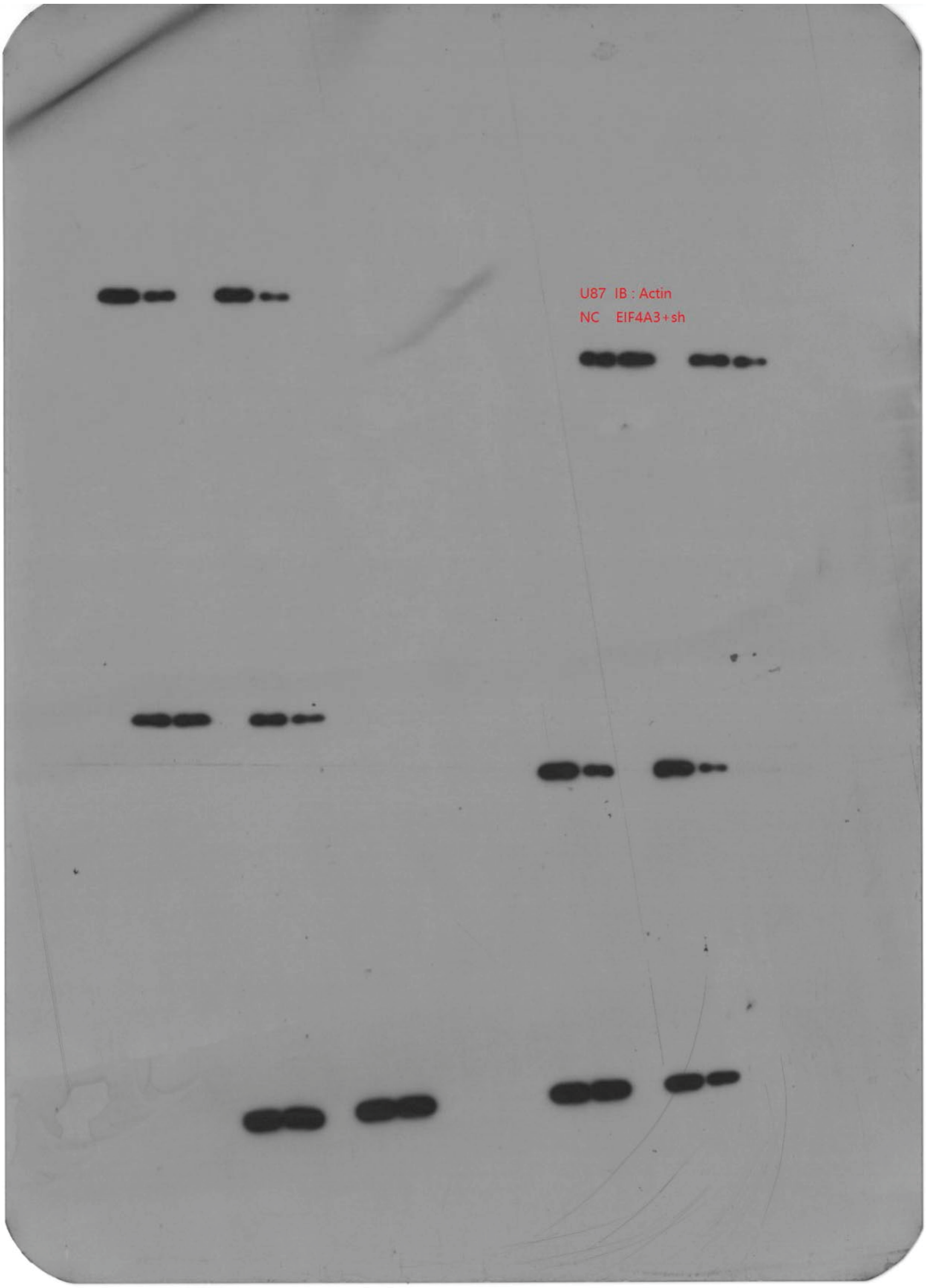

Figure 4a-T98G-Actin

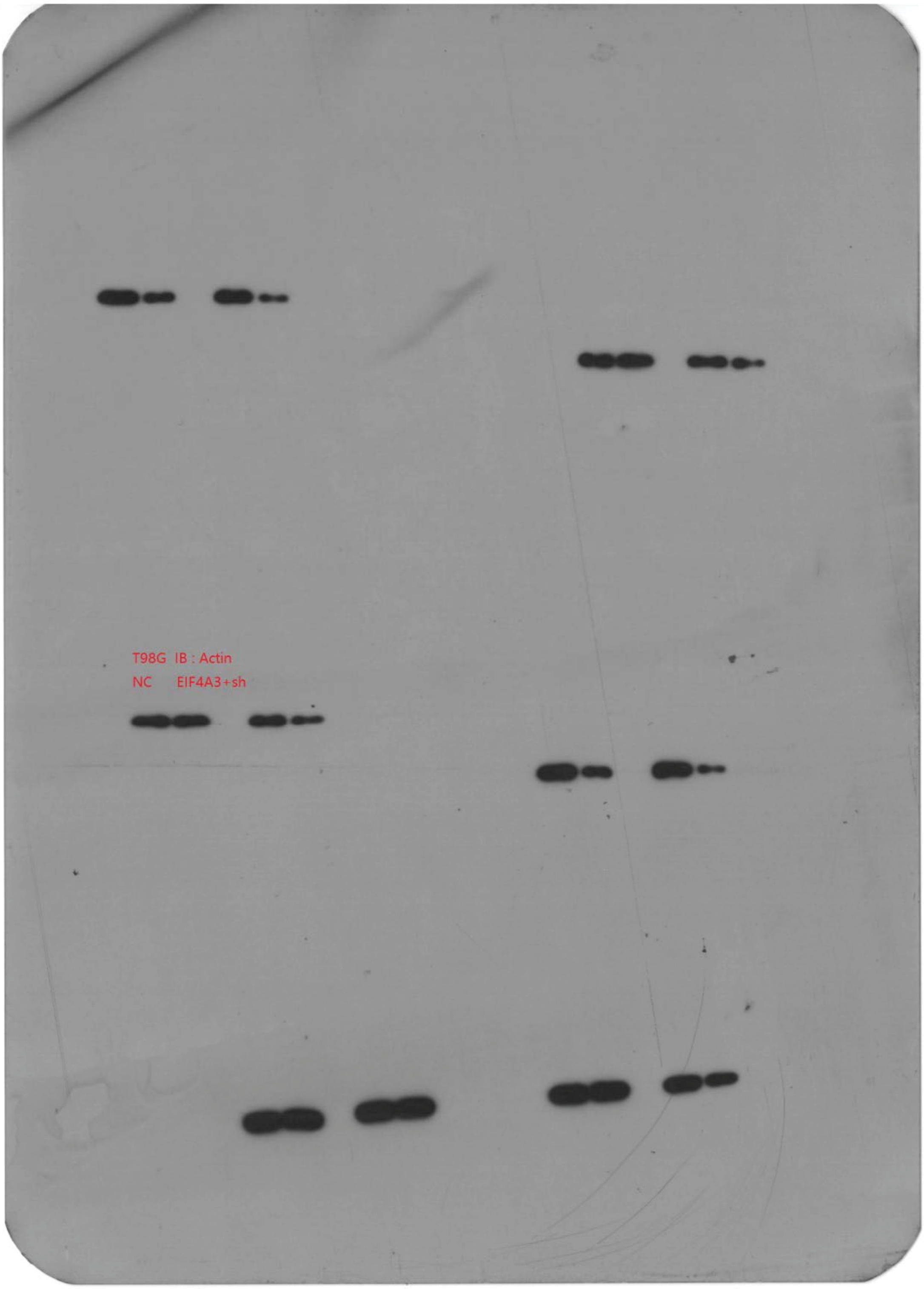

Figure S1aU251, IB: EIF4A3

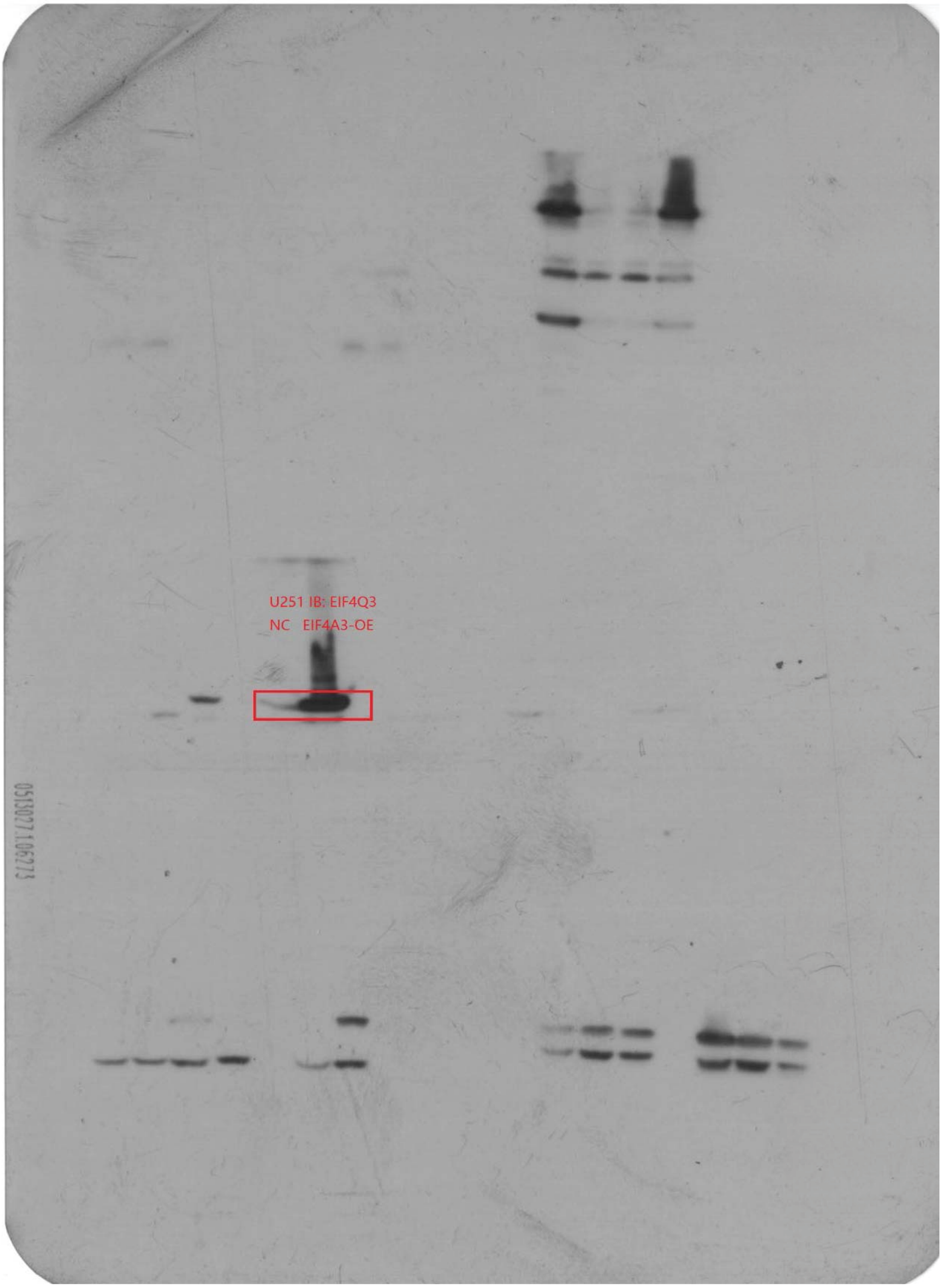

Figure S1a A172, IB: EIF4A3

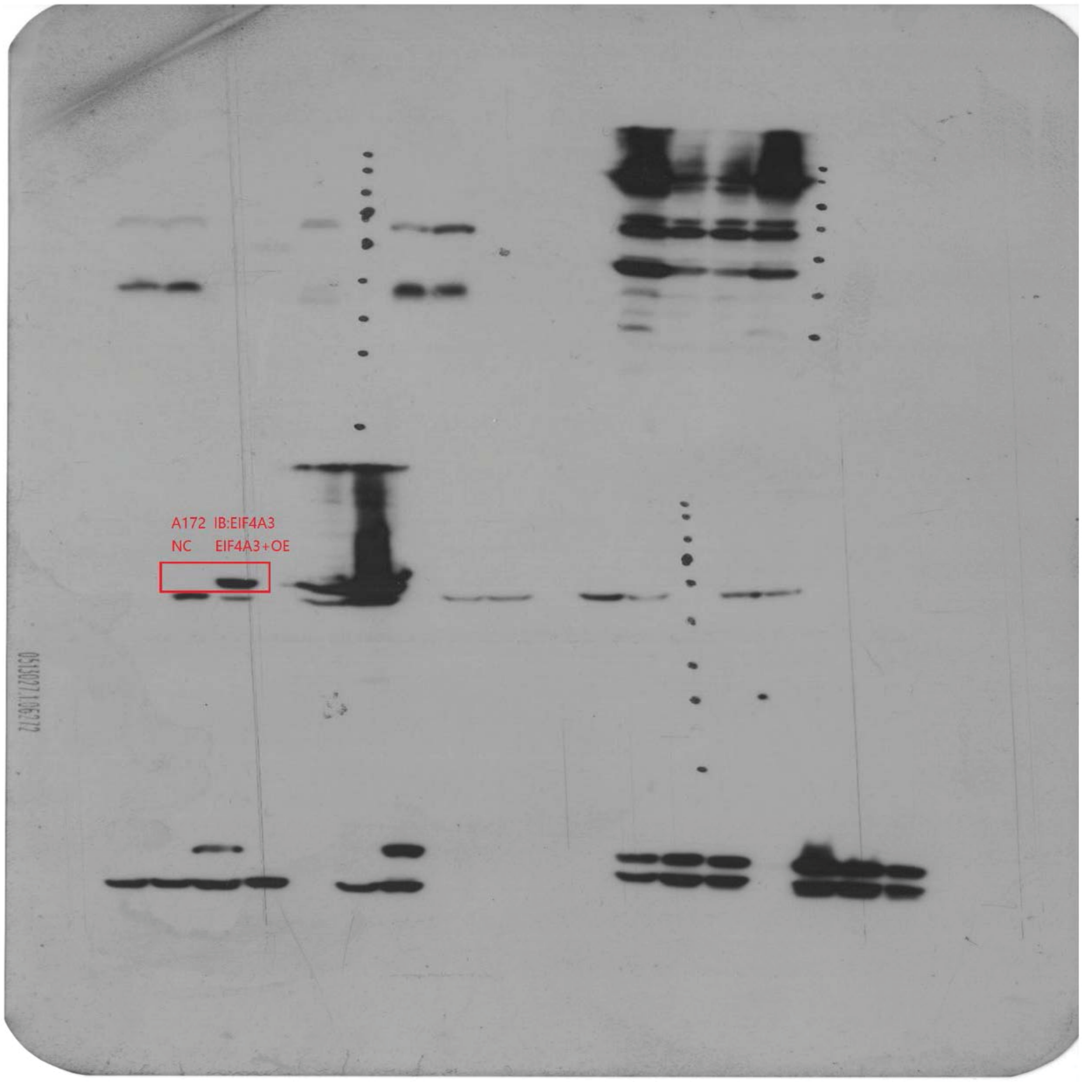

Figure S1a-A172-U251, IB: Actin

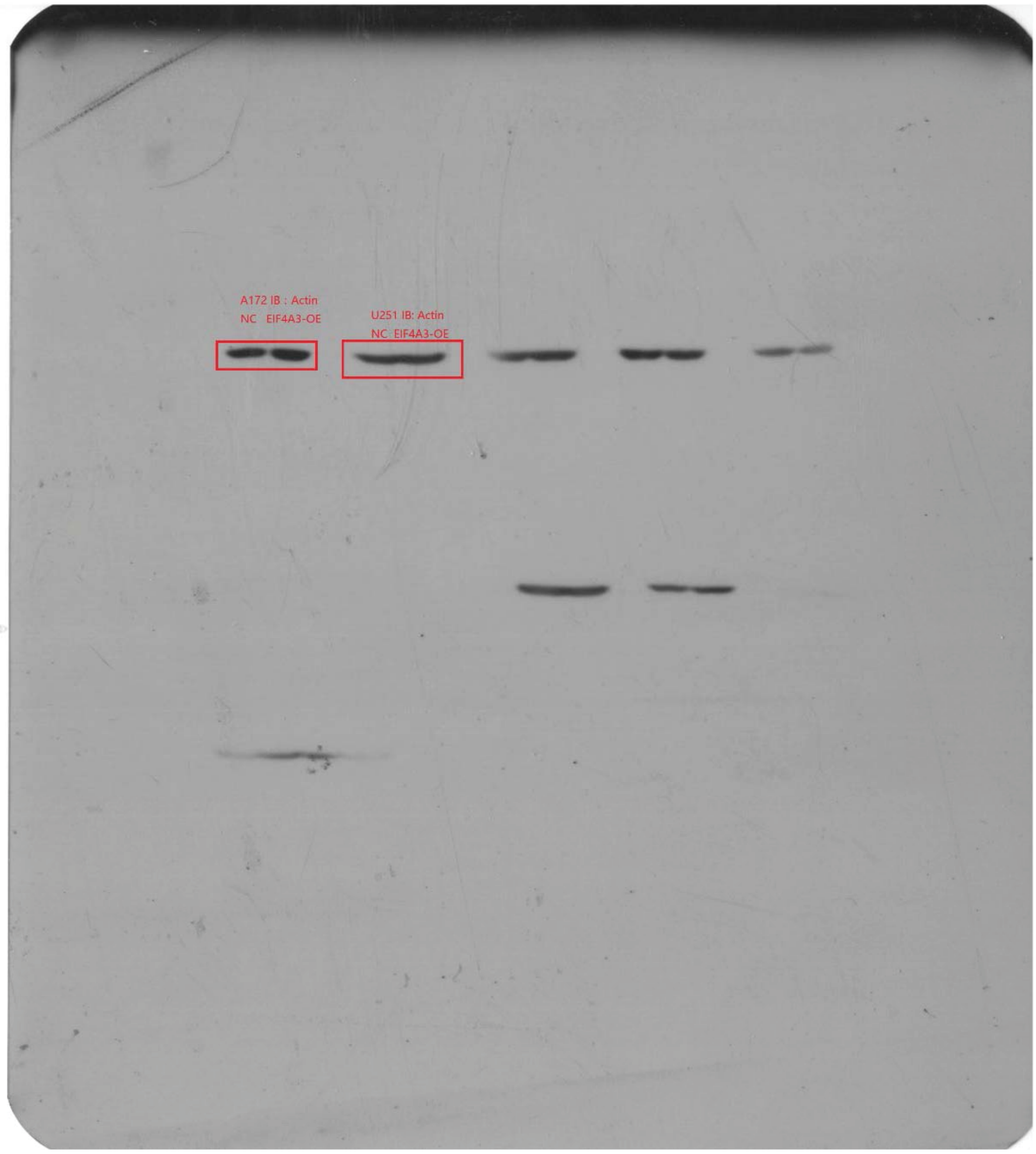

Figure 5s, IB: EIF4A3

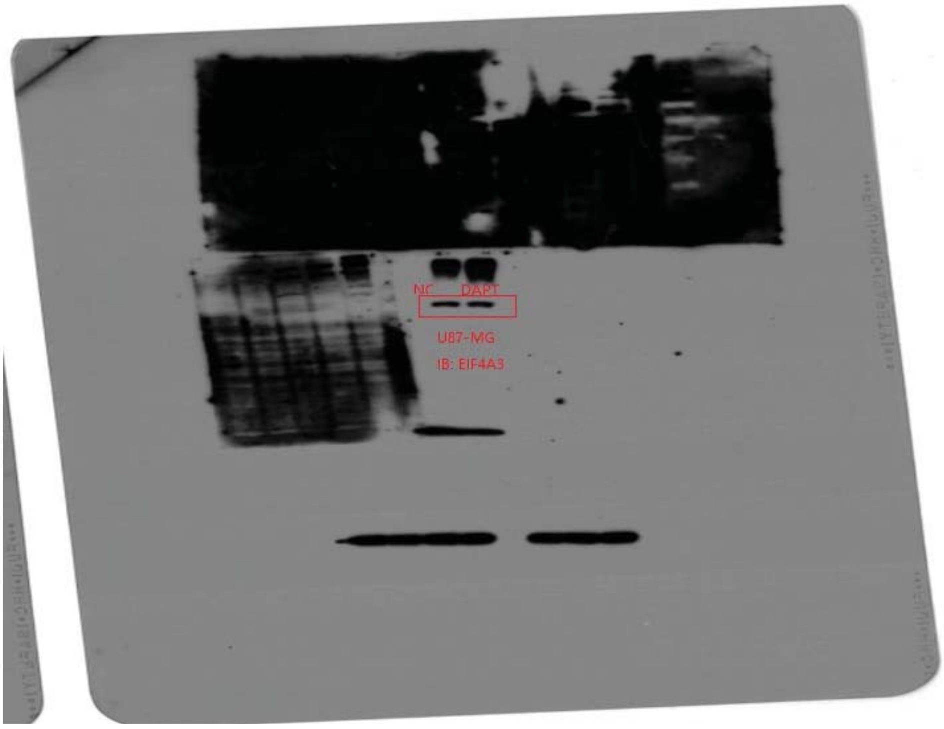

Figure 5s, IB: Actin

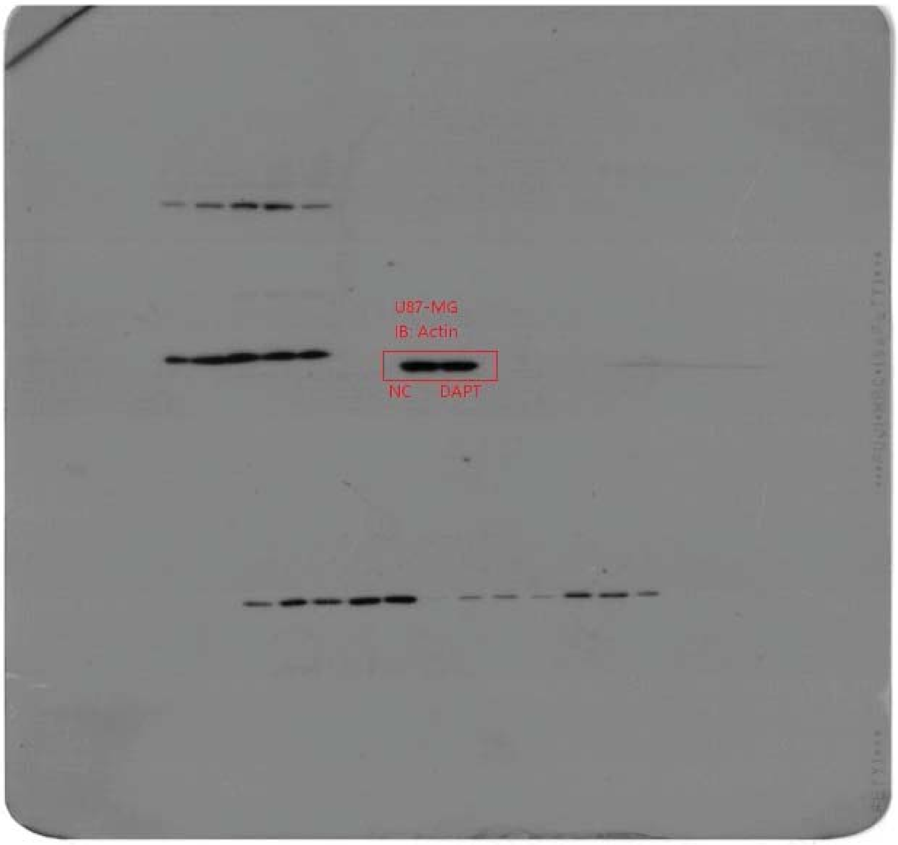

Supplement: Supplementary file 2 — Supplementary Material 2 [file 12885_2023_10946_MOESM2_ESM.pdf]
